# Pyrotinib targeted EGFR/GRP78 mediated cell apoptosis in high EGFR gene copy number gastric cancer

**DOI:** 10.1186/s13046-025-03485-6

**Published:** 2025-08-19

**Authors:** Lingbo Bao, Xudong Wang, Xiuyong Liao, Dong Li, ChunXue Li, Nan Dai, Xiaoyan Dai, Jing Yang, Nana Hu, Xueling Tong, Zhenjie He, Yuancheng Zhao, Zheng Liu, Yue Hu, Jinlu Shan, Dong Wang, Mengxia Li, Qian Chen

**Affiliations:** 1 Cancer Center of Daping Hospital, Army Medical University, Chongqing, 400037 China; 2 Department of Oncology, Chongqing University Qianjiang Hospital, Qianjiang Central Hospital of Chongqing, No. 360, South Section of Zhengzhou Road, Qianjiang District, Chongqing, 409000 China; 3 Department of Oncology, General Hospital of Western Theater Command, Chengdu, 610083 China; 4 Department of General Surgery, Daping Hospital, Third Military Medical University (Army Medical University), Chongqing, 400042 China; 5 Department of Ophthalmology, The General Hospital of Western Theater Command, Chengdu, 610083 China

**Keywords:** Pyrotinib, EGFR, GRP78, Ubiquitination, TRIM21

## Abstract

**Background:**

Despite frequent Epidermal Growth Factor Receptor (*EGFR*) amplification and overexpression in gastric cancer, limited therapeutic responses were observed in existing EGFR-targeted agents. Pyrotinib, an irreversible dual EGFR/HER2 tyrosine kinase inhibitor, has shown clinical efficacy in HER2-driven malignancies, but its potential role in *EGFR*-high copy number gastric cancer remains to be investigated.

**Methods:**

Using *EGFR*-high copy number gastric cancer cell lines, primary cells and subcutaneous tumor models in nude mice, we systematically evaluated pyrotinib’s anti-tumor activity through viability assays, apoptosis analysis, and transcriptomic profiling. Mechanistic studies included co-immunoprecipitation, proximity ligation assays, ubiquitination assays, and RNA sequencing.

**Results:**

Pyrotinib selectively suppressed proliferation, induced apoptosis, and chemosensitized in *EGFR*-high copy number gastric cancer models. Mechanistically, pyrotinib promoted EGFR-GRP78 (Glucose-regulated protein 78) complex formation in the endoplasmic reticulum, activating the protein kinase R-like endoplasmic reticulum kinase/ activating transcription factor 4/ C-EBP homologous protein (PERK/ATF4/CHOP) axis to drive ER stress-mediated apoptosis. Concurrently, pyrotinib inhibited GRP78 phosphorylation at Thr62, triggering K48-linked ubiquitination (ubiquitin chains formed via lysine 48 linkages) and proteasomal degradation, which impaired DNA double-strand break (DSB) repair and sensitized cells to oxaliplatin-induced γ-H2A.X accumulation.

**Conclusion:**

This translational study suggests that pyrotinib combined with oxaliplatin may serve as a promising strategy for patients with *EGFR*-high copy number gastric cancer and highlighted the discovery of this previously unknown EGFR/ GRP78 signaling axis, which provides the molecular basis and the rationale to target EGFR.

**Supplementary Information:**

The online version contains supplementary material available at 10.1186/s13046-025-03485-6.

## Introduction

Gastric cancer (GC) is a prevailing malignant tumor globally, ranking fifth in incidence and mortality rates among all cancer types as reported by GLOBOCAN 2022 [1]. While targeted therapies have revolutionized cancer management, durable responses in GC remain limited. Since the landmark ToGA trial established trastuzumab, the anti-HER2 antibody, as first-line therapy for *HER2*-positive advanced gastric cancer [2], research has expanded to other targets including human epidermal growth factor receptor (EGFR), vascular endothelial growth factor (VEGF) [3], and Claudin 18.2 [4]. Nevertheless, not all patients respond to targeted therapy, underscoring the urgent need for novel targeted agents for GC to improve the clinical outcomes.

The HER (human epidermal growth factor receptor) family, comprising EGFR/HER1, HER2, ErbB3/HER3, and ErbB4/HER4, plays a pivotal role in GC through ligand-independent homodimerization and subsequent activation of oncogenic signaling pathways [5]. Clinically, *HER* amplification and protein overexpression correlate with aggressive tumor behavior, including poor prognosis and therapeutic resistance in GC. Among these receptors, EGFR and HER2 have emerged as prime therapeutic targets, with their tyrosine kinase domains constituting key molecular sites for pharmacological intervention [6]. Malignant phenotype and poor prognosis, which is associated with activation of EGFR downstream signaling, occurred in multiple malignancies, specifically NSCLC, GC, glioma, and colorectal cancer, etc [7]. GC subtype with EGFR amplification and overexpression benefit from anti-EGFR treatment [8]. Despite the importance of the HER family in the tumorigenesis of GC, anti-EGFR therapies (e.g., cetuximab, panitumumab, gefitinib) have shown limited success in GC clinical trials [9, 10]. Emerging evidence suggests pan-HER inhibitors may offer superior anti-tumor activity in preclinical GC models [11, 12], suggesting that pan-HER inhibitors may be a more efficacious therapeutic strategy in GC.

Mechanistically, many anti-tumor agents impede cell proliferation and induce cell apoptosis by triggering endoplasmic reticulum (ER) stress [13]. ER stress signaling also facilitates the survival of cancer cells tolerant to EGFR tyrosine kinase inhibitors [14], and mediates radioresistance subsequent to EGFR inhibition by cetuximab [15]. GRP78, a key ER protein, coordinates protein import, folding and ER-associated protein degradation by evoking endoplasmic reticulum stress under stress conditions like sugar deprivation, treatment with inhibitor of protein glycosylation or intracellular calcium storage disturbances [16]. Post-translational modifications of GRP78 including phosphorylation [17] and ubiquitination [18] regulate its basic function. GRP78 has a KDEL (Lys-Asp-Glu-Leu) sequence at its C-terminus, which signals its retention in the ER. GRP78 is post-translationally modified by phosphorylation on serine residues in the ER [19]. The phosphorylation of GRP78 may be converted into functional forms by dephosphorylation under starvation [20]. GRP78 phosphorylation also affects its ubiquitination [21], which is important in the progression of cancer [22].

In our research, we screened a panel of HER inhibitors and identified pyrotinib as a potent HER inhibitor that selectively suppresses the growth of GC with high EGFR gene copy number (CN) in vitro and in vivo. The combination of pyrotinib with oxaliplatin demonstrated synergistic therapeutic potential in GC patients with *EGFR*-high CN. Mechanistic investigations revealed pyrotinib promoted EGFR-GRP78 complex formation in the ER lumen, subsequently activating the PERK/ATF4/CHOP signaling pathway, and finally resulted in tumor apoptosis. Additionally, pyrotinib sensitized GC cells to oxaliplatin by through the inhibition of GRP78 phosphorylation at Thr62, resulting in GRP78 ubiquitination. These findings establish the EGFR/GRP78 axis as a critical mediator of pyrotinib-induced apoptosis, and position this agent as a promising therapeutic for *EGFR*-hyperactive GC.

## Materials and methods

### Human cell lines and primary cell culture

SGC7901, MGC803, MKN45, SNU5, SNU601, NUGC4, SNU719, AGS, NCI-N87, HEK293T were purchased from Pricella (Wuhan, China). AGS cells were cultured in F12 medium containing 10% FBS, and other cell lines were cultured in RPMI 1640 supplemented with 10% FBS.

For primary culture of GC, fresh cancerous tissue was obtained from informed patients who had undergone surgical resection of primary GC and cut into pieces. The single cell suspension was digested with a mixture of collagenase I and IV. The primary cultures were established in RPMI 1640 medium containing 10% FBS. The successfully established primary GC cells was designated GC-1 and GC-2. All gastric cells were maintained at 37 ℃ with 5% CO_2_ incubator.

### Reagents and antibodies

Antibodies against rabbit PERK (#3192), rabbit GRP78 (for western blot, #3177), rabbit eIF2α (#5324), rabbit p-eIF2α (#3398), rabbit HER family antibody sampler kit (#8339), mouse Cleaved Caspase-9 (Asp353; #9509), rabbit HA-Tag (#3724), rabbit DYKDDDDK Tag (#14793), rabbit HIS (#2365), rabbit AKT (#9272), rabbit p-AKT (#4060) and rabbit GAPDH (#2118) were purchased from Cell Signaling Technology. Antibodies against rabbit ATF4 (ab270980), rabbit p-EGFR (phospho Y1068; ab40815), rabbit γ-H2A.X (ab81299) and rabbit p-(Ser/Thr)Phe (ab300625) were obtained from Abcam. Antibodies against rabbit CHOP (15204-1-AP), mouse GRP78 (for immunofluorecence, 66574-1-Ig) were purchased from Proteintech. Anti-FLAG M2 Magnetic Beads (M8823) were got from Sigma-Aldrich. Dacomotinib (HY-13272), afatinib (HY-10261), pyrotinib (HY-104065), oxaliplatin (HY-17371), MG132 (HY-13259), SAL003 (HY15869) were purchased from MedChemExpress (New Jersey, USA) respectively. Cycloheximide (CHX) was purchased from Selleck (S7418). Leupeptin was purchased from Beyotime (SG2012).

### Cell viability assay and synergy assay

Cells were seeded into 96-well plates and exposed to different concentrations of pan-HER TKIs (Pyrotinib, Afatinib, Dacomotinib) and Oxaliplatin for indicated times. Next, 100 µL of CCK-8 (C0037, Beyotime) reagent were incubated at 37 °C for 2 h, and measured the absorbance at 450 nm. For dose-response curves, cells were plated at 20–40% confluence and treated for 48 h with the drugs dissolved in DMSO.

Drug synergy was assessed with Combenefit software [23]. Cells were treated with drug combinations in a 5-by-5 drug concentration matrix and then the cell viability was evaluated. Bliss synergy scores were calculated for each drug combination using Combenefit software (v2.021).

### *In vivo* study

Animal experiments were approved by the Ethics Committee of Third Military Medical University. Female nude mice (4–6 weeks old) were housed in the laboratory of animal center, Daping hospital, Third Military Medical University. SNU719 (2 × 10^6^), NUGC4 (2 × 10^6^), SGC7901 (1 × 10^6^) cells were suspended in mixture of 0.1 mL Matrigel (Corning) and 0.1 mL of PBS, and were injected subcutaneously into the groin of the mice to establish a tumor xenograft model. When the tumor volume reached 100 mm^3^, mice were randomly divided into several groups. Pyrotinib (acquired from Hengrui Pharmaceuticals Co.) was administered orally at a dose of 10 mg/kg daily, and/or oxaliplatin was given intraperitoneally twice a week at 5 mg/kg, and the control group was treated with phosphate-buffered saline (PBS). Body weight and tumor size were measured every 2 days. Tumor volume was calculated using the formula: tumor volume = (width^2^ × length)/2. After 12 days, the mice were killed and examined by immunohistochemistry (IHC). Sample size of animal experiments were testified by power analysis.

For testing the tumorigenicity of SNU719 pyrotinib-resistant (SNU719-Pyr^R^) cells, 2 × 10^6^ cells were suspended in mixture of PBS/Matrigel (1:1 v/v), and injected subcutaneously into the groin of the mice. At day 7, mice were randomly divided into four groups. Pyrotinib was given 10 mg/kg orally once a day, and/or SAL003 were intratumorally injected 1.5 mg/kg/day. The xenograft tumors were detected and quantified by bioluminescence imaging using In Vivo Image System (IVIS spectrum in vivo imaging system) at 21 days. And then, the mice were killed and examined by immunohistochemistry (IHC).

### Next-generation DNA sequencing and transcriptomic sequencing

Next-generation DNA sequencing and transcriptomic sequencing were performed and analyzed by ZhenHe Bioinformatics Institute Co. (Wuxi, China) as previous study [24]. And for DNA sequencing, we provide a quantitative threshold for defining “high” EGFR CN as CN ≥ 5 [25].

For transcriptomic profiling, differential gene expression analysis of RNA-seq raw counts was performed using the DESeq2 package (v1.38.3). Significant differentially expressed genes (DEGs) were identified with thresholds of|log2FoldChange| >1 and adjusted *p*-value (*p*.adj) < 0.05. Gene Ontology (GO) enrichment analysis was conducted via the clusterProfiler package (v4.6.2), with gene ID conversion using “org.Hs.eg.db” (v3.17.0). Top 20 enriched terms ranked by Rich factor (gene count normalized to background) were visualized.

Gene Set Enrichment Analysis (GSEA) was performed using the clusterProfiler package (v4.6.2). Gene sets were retrieved from the MSigDB C5 ontology (v2024.1, GO biological processes) and mapped to gene symbols. Enrichment scores were calculated using the GSEA algorithm, with significance evaluated via 1000 permutations. Leading-edge gene sets (adjusted *p*-value < 0.05) were prioritized. Visualization of enriched pathways (e.g., “*PERK*-mediated unfolded protein response”) and key annotated genes (e.g., *ATF4*) was implemented via the “enrichplot” package, employing running score plots and label-based gene highlighting. Analytical parameters and workflows are detailed in Supplementary Materials.

### Plasmid constructs, virus production and cell infection

Plasmid constructs for the ectopic expression of full-length EGFR (pCMV3-EGFR-FL-HA), extracellular domain deletion variant (pCMV3-EGFR-ΔECD-HA), tyrosine kinase domain deletion variant (pCMV3-EGFR-ΔTKD-HA), C-terminal deletion variant (pCMV3-EGFR-ΔC-HA), full-length GRP78 (pCMV3-GRP78-FL-Flag), and other GRP78 mutants, pCMV3-PERK, pCMV3-control-Flag, pCMV3-Ub-his, pCMV3-Ub-K48O-His, and pCMV3-Ub-K63O-His were all obtained from Sino Biological (Beijing). NUGC4, SNU719 and HEK293T were transfected with plasmid using Lipofectamine 3000 (Thermo Fisher Scientific) according to the manufacturer’s instructions.

To generate stable EGFR and ATF4 knockdown GC cells, SNU719 and NUGC4 (1 × 10^6^ /well) were transfected with lentiviral vector (Genechem, Shanghai, China) carrying self-complementary hairpin DNA fragments that could generate EGFR or ATF4-specific shRNA, or non-targeting scrambled RNA (shNC). Cells were infected with lentivirus supplemented with 8 mg/ml polybrene (Sigma-Aldrich) and then selected with 5 µg/mL puromycin (MedChemExpress) for 7 days. The shRNA sequences are listed in Supplementary Table 1.

### RNA interference

NUGC4 or SNU719 GC cells were seeded in six-well plates (1 × 10^5^) and transfected with si*RNA* using Lipofectamine 3000 in Opti-MEM medium for 6 h. Then, the medium was replaced with fresh RPMI 1640 medium containing 10% FBS. After another 96 h culture, indicated cells were harvested and analyzed protein expression by western blotting. The siRNA for EGFR (sc-29301), HER2 (sc-29405) and HER4 (sc-35329) were acquired from Santa Cruz. The *GRP78* si*RNA* was acquired from Genepharma and the sequences are listed in Supplementary Table 2.

### Cell cycle analysis

For cell cycle arrest assay, NUGC4 and SNU719 cells were subjected to treatment with pyrotinib (1 µM), oxaliplatin (1 µg/mL), and their combinations for 48 h. Subsequently, the treated cells were harvested and washed twice with PBS, and then fixed at -20℃ with 70% precooled ethanol for 1 h. Finally, the cells were stained with propidium iodide (PI) at 4 °C for another 30 min and measured by flow cytometer (CytoFLEX, Beckman Coulter).

### Apoptosis analysis

SGC7901, NUGC4 and SNU719 cells (5 × 10^5^ cells/well) were seeded in 6-well plates for the apoptosis assay and exposed to DMSO or Pyr (1 µM) for 48 h. Subsequently, the cells were washed with PBS and processed with the Annexin V-FITC/PI apoptosis detection kit (C1052, Beyotime) in accordance with the protocol. Finally, the cells were detected by the flow cytometer (CytoFLEX, Beckman Coulter), and the analysis was performed using Flowjo software.

### EdU cell proliferation assay

The EdU cell proliferation assay was conducted in accordance with the manufacturer’s protocol. 10 µM EdU reagent (C0071S, Beyotime) was added to the cells treated with different drugs and incubated for 2 h. After three washes with PBS, the cells were fixed with 4% paraformaldehyde solution for 15 min, permeabilized with 0.3% Triton X-100 (P0096, Beyotime) for another 15 min, and then incubated with the click-reaction reagent for 30 min at room temperature in the dark. Finally, 1× Hoechst 33,342 reagent was used to counterstain the nucleus.

### Measurement of ROS

Cells were seeded in six-well plates and treated with DMSO or different concentrations of pyrotinib (0.5, 1, and 2 µM). Subsequently, cells were incubated with DCFH-DA (diluted 1:1000 in serum-free RPMI 1640, BB-47049, Bestbio) at 37 °C for 30 min and washed three times with the medium. The level of ROS was determined by flow cytometer.

### Measurement of intracellular calcium ion

The intracellular calcium ion levels were determined using the diluted Fluo-4 AM fluorescent probe (S1060, Beyotime). SNU719 cells were exposed to DMSO or different concentrations of pyrotinib (0.5, 1, and 2 µM), washed three times with PBS, and subsequently incubated with the Fluo-4 AM working solution (2 µM, 1:1000 diluted in PBS) at 37 °C for 30 min. After being washed three times with PBS, cells were further incubated for another 30 min. Eventually, the calcium ion concentration was detected by flow cytometer using FITC channel.

### Western blotting and immunoprecipitation

Cells harvested from cultured dishes subjected to different treatments were lysed using RIPA lysis buffer supplemented with 1 mM Phenylmethanesulfonyl fluoride (PMSF, ST506, Beyotime) and 1% phosphatase inhibitor cocktail I (HY-K0021, MCE) and II (HY-k0023, MCE). The protein concentration was ascertained with the BCA protein Assay Kit (P0009, Beyotime). Total protein (30 µg) was separated by 4–20% FuturePAGE (ET15420LGel, ACE), and transferred with 0.22 µM polyvinylidene fluoride (PVDF) membrane (Millipore), blocked with 5% (w/v) skim milk/TBST at room temperature for 1 h, and incubated with primary antibodies overnight. Subsequently, secondary antibodies were incubated at 37 °C for 1 h. Finally, the membranes were washed with TBST and the densitometric values were determined by gel image analysis system (Bio-Rad).

Co-IP was performed as previously study [26]. Briefly, cells in 6 cm cell culture dishes were transfected with the appropriate plasmids, which were extracted using Pierce IP lysis buffer (87787, Thermo Fisher) along with protease inhibitors. The IP lysates were captured with the appropriate anti-Flag M2 magnetic beads (M8823, Sigma-Aldrich) overnight at 4 °C. After being washed three times with PBS, the beads were boiled with SDS-PAGE loading buffer for 10 min and then further subjected to immunoblotting.

### Immunohistochemistry

Subcutaneous tumors were collected and fixed with 4% paraformaldehyde fixative. Subsequently, the tissues were paraffin-embedded and sectioned at 3 μm. The deparaffinized and rehydrated sections underwent antigen retrieval using sodium citrate buffer. After being incubated with 5% goat serum for 1 h, the sections were incubated with the primary antibody overnight at 4 °C. Then, the sections were washed and incubated with MaxVision HRP-Polymer anti-Mouse/Rabbit IHC Kit (KIT-5030, Maxim) for 30 min at 37 °C. The color was developed using 3,3′-diaminobenzidine (DAB) (P0202, Beyotime).

### Proximity ligation assay

For the proximity ligation assay, cells were seeded onto glass coverslips and fixed with 4% paraformaldehyde. The cells were washed with PBS and permeabilized with 0.1% Triton X-100 in PBS for 20 min. After blocking, the cells were incubated overnight at 4 °C with anti-EGFR (#4267, Cell Signal Technology) and anti-GRP78 antibodies (66574-1-Ig, Proteintech), which were diluted in Duolink Antibody diluent (DUO82008, Sigma Aldrich). Subsequently, the prediluted PLUS (DUO92002, Sigma Aldrich) and MINUS probes (DUO92004, Sigma Aldrich) were incubated at 37 °C for 1 h. Then, cells were incubated with 1× ligase at 37 °C for 30 min and continued with 1× polymerase at 37 °C for 100 min for amplification. Finally, the slides were mounted with Duolink in situ mounting medium with DAPI (DUO82040, Sigma Aldrich).

### TUNEL assay

TUNEL assay was conducted as described previously using the TUNEL apoptosis detection kit (BA2020, Enogene) (39389211). Briefly, the dewaxed sections were subjected to treatment with proteinase K and blocked by 3% H2O2 in methanol. Subsequently, they were reacted with TdT enzyme reaction buffer at 37 °C in the dark for 60 min, followed by incubation with Streptavidin-HRP for 30 min. Finally, the sections were mounted using a mounting medium containing DAPI.

### Immunofluorescence

Immunofluorescence was conducted on slices. Initially, the cells were treated by 1 µM ER-tracker red (Beyotime, C1041S) with or without Dio (Beyotime, C1038) at 37 °C for 20 min, followed by fixation with 4% paraformaldehyde for 5 min and permeabilization with 0.1% Triton X-100 for 10 min. After blocking with 5% goat serum for 30 min, the cells were incubated with rabbit anti-EGFR (#4267, Cell Signal Technology) and mouse anti-GRP78 (66574-1-Ig, Proteintech) antibodies at 4℃ overnight. Then incubated with Alexa Fluor 647-labeled goat anti-rabbit IgG (H + L) (A04268, Beyotime) and Alexa Fluor 488-labeled goat anti-mouse IgG (H + L) (A0428, Beyotime) at room temperature for 2 h, and finally incubated with DAPI for 10 min. The slides were observed under a confocal scanning microscope.

### Fluorescence in-situ hybridization

Fluorescence in-situ hybridization (FISH) was performed in accordance with the manufacturer’s protocol. For pretreatment, the tumor slides were dewaxed and dehydrated. Firstly, 10 µL EGFR/CEN7 FISH probe (FG0242, Abnova) was applied and then denatured at 75℃ for 5 min. Subsequently, hybridization incubation was conducted at 37℃ in humidified hybridization chamber for 24 h. Next, the washing procedure was carried out by sliding the section into 2x SSC, and successively incubated for 5 min at room temperature (RT), 2 min at 73℃, and 1 min at RT. Finally, the sections were mounted with the mounting medium containing DAPI. The average *EGFR* CN per cell was calculated by the *EGFR/CEN7* ratio. The *EGFR/CEN7* ratio for each evaluable tissue core was calculated by dividing the total number of *EGFR* gene signals by the total number of chromosome 7 centromere (*CEN7*) signals counted across all evaluable tumor cells within that core. *EGFR*-high CN was defined as an *EGFR/CEN7* ratio ≥ 1.5, and the other tumors were classified as *EGFR*-low. Cancer tissue cores that had no more than 1 signal for either EGFR or CEN7 per cell were excluded from further analyses [27].

### Statistical analysis

All experimental data were analyzed using GraphPad Prism 9.0 and R 4.3.1. Normality was assessed via Shapiro-Wilk test; parametric tests (Student’s *t*-test or ANOVA with Tukey’s post-hoc) or non-parametric equivalents (Mann-Whitney U or Kruskal-Wallis) were applied accordingly. Data are presented as mean ± SEM (*n* ≥ 3 biological replicates), with *p* < 0.05 considered significant. Multiple comparisons were adjusted via Benjamini-Hochberg false discovery rate (FDR). Paired Student’s *t*-tests were applied to evaluate differences between paired measurements (e.g., different transfection plasmids) under the same experimental conditions.

### Database on line and bioinformatics analysis

The correlation between *EGFR* copy number and pyrotinib sensitivity in GC cell lines was analyzed using the DepMap database (release 2024Q2). Cell lines were filtered to include only *HER2 (-)* subtypes based on genomic annotations. *EGFR* CN data were extracted logarithmically, and pyrotinib sensitivity was quantified log fold change (LFC) to control. Pearson correlation analysis was performed to assess associations. Results were visualized using scatter plots with regression lines, highlighting *EGFR* CN trends relative to drug response. Publicly accessible data and tools from DepMap (https://depmap.org) were utilized for this study.

### Molecular docking procedures

Protein structures of EGFR (P00533) and GRP78 (P11021) were retrieved from the UniProt database [28]. The 3D structure of Pyrotinib (51039030) was obtained from PubChem. Primary docking between EGFR and Pyrotinib was performed using CB-DOCK2 [29]. Subsequently, the pre-docked EGFR-Pyrotinib complex was rigidly docked to GRP78 using the GRAMM web server (https://gramm.compbio.ku.edu/) [30].

## Results

### High *EGFR* gene copy number predicts the therapeutic efficacy of pyrotinib in gastric cancer

**Fig. 1:**
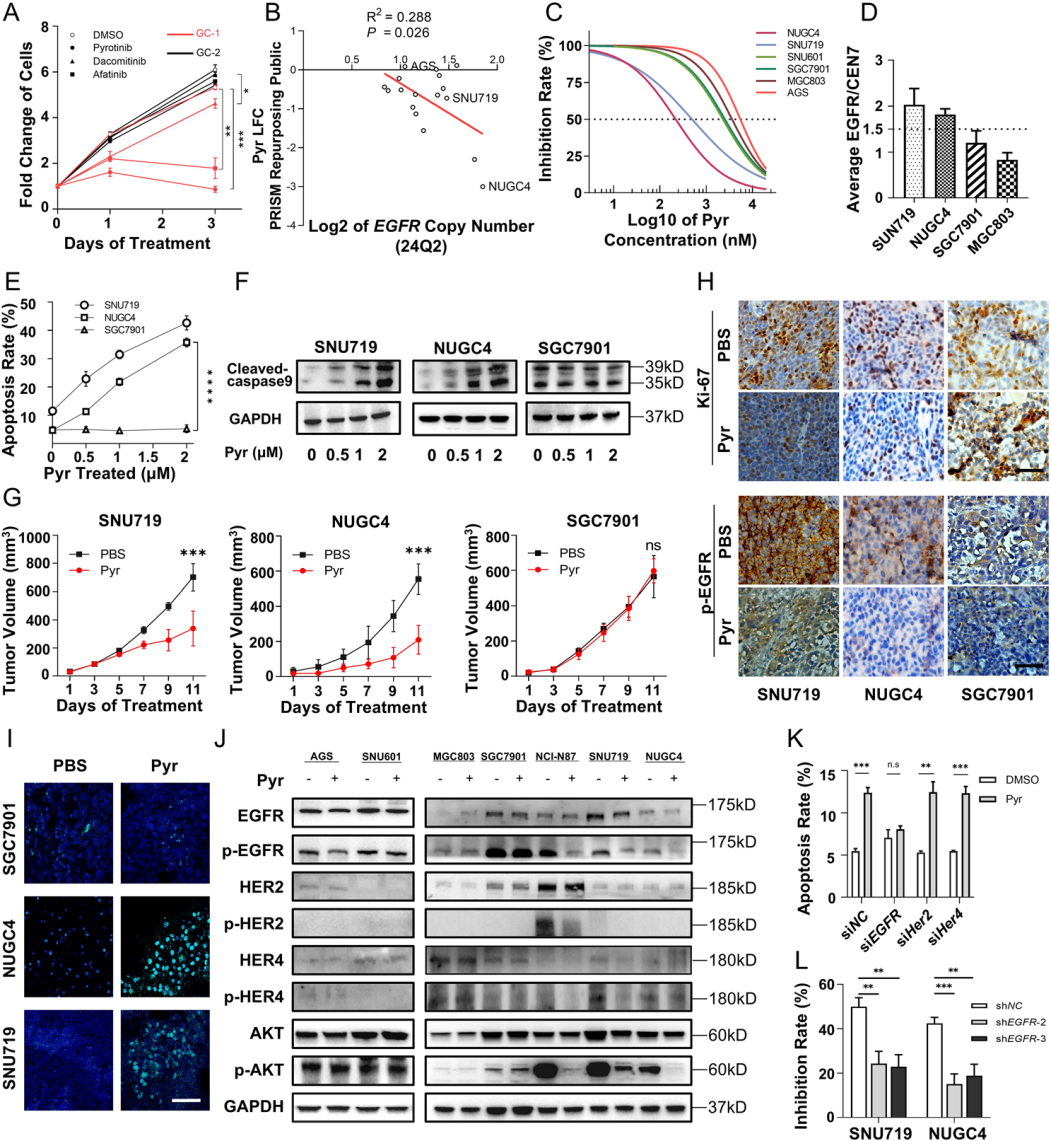
Pyrotinib suppresses *EGFR*-high copy number gastric cancer through inhibition of EGFR signaling. **A**. Primary gastric cancer (GC-1 and GC-2) cells treated with pyrotinib, dacomitinib, afatinib, or DMSO (control) at 1 µM concentration over 3 days, growth curves represent three independent experiments. **B**. Correlation analysis between pyrotinib sensitivity (log fold change, LFC) and *EGFR* gene copy number in a set of 17 HER2 negative GC cell lines from DepMap database. *p* = 0.026, R^2^ = 0.288. **C**. Cell growth of 6 GC cell lines was measured after exposure to indicated concentrations of pyrotinib (0, 10, 100, 1000, 10000 nM) for 72 h using CCK-8 assay. **D**. *EGFR* copy number in four gastric carcinoma induced xenograft was detected by fluorescence in situ hybridization. **E**. GC cells were harvested and stained with Annexin V-FITC and PI for apoptotic analysis with increasing concentrations of pyrotinib treatment for 48 h. **F**. Western blotting analysis showed the cleaved caspase-9 level in GC cells treated with different dose of pyrotinib. **G-I**. GC cells were subcutaneously implanted into nude mice. After 10 days, PBS or pyrotinib was orally administered into the mice respectively (*n* = 5). Tumor specimens were collected from sacrificed mice on day 11 after administration. (**G**) Tumor volume of indicated xenograft models treated with PBS or Pyr were displayed by growth curves. (**H**) Representative immunohistochemical staining of Ki-67 and phosphorylated EGFR (p-EGFR) in xenograft tumor tissues excised from indicated mice. Bars, 100 μm. (**I**) Representative TUNEL immunostaining (green) of apoptosis in xenograft tumor tissues excised from indicated mice. Bars, 100 μm. **J**. Western blot analysis of EGFR, HER2, HER4, and downstream signaling (AKT and p-AKT) in 7 GC cell lines treated with pyrotinib or DMSO. HER2 high CN: NCI-N87; HER2 low CN and *EGFR* high CN: NUGC4 and SNU719; HER-2 low CN and EGFR low CN: AGS, SNU601, MGC803, SGC7901. **K**. GC cells transfected with siRNAs targeting *EGFR*, HER2, or HER4 were treated with 1 µM pyrotinib for 48 h. Cells were harvested and stained with Annexin V-FITC/PI for apoptosis analysis. **L**. Sensitivity of NUGC4 and SNU719 (transfected with sh*NC*, sh*EGFR-2* or sh*EGFR-3*) to pyrotinib (1 µM, 24 h) were detected by CCK-8 assay. Significance was determined by Paired t-test (A, E, G) and One-way ANOVA (K, L). *p < 0.05; **p < 0.01; ***p < 0.001

To investigate the growth-inhibitory effects of pan-*HER* inhibitors on GC cells, two primary GC cell lines (GC-1 and GC-2) were established, and gene CN alterations were analyzed by next-generation sequencing (NGS). Notably, treatment with pan-HER inhibitors (pyrotinib, dacomitinib, afatinib) significantly suppressed cell proliferation in GC-1 but showed no inhibitory effect on GC-2 (Fig. 1A). Comprehensive genomic profiling by NGS revealed both cell lines were *HER2/HER3/HER4*-negative metastatic GC models, while EGFR CN analysis identified GC-1 with high *EGFR* CN and GC-2 with low CN (Fig. S1A), suggesting a correlation between *EGFR* status and pan-HER inhibitor sensitivity. Among the tested inhibitors, pyrotinib demonstrated superior anti-proliferative activity across multiple concentrations compared to dacomitinib and afatinib (Fig. S1B). Consistent with this, DepMap database analysis revealed a positive correlation between pyrotinib sensitivity and *EGFR* CN in 17 *HER2/HER3/HER4*-negative GC cell lines (Fig. 1B).

To validate these findings, pyrotinib efficacy was assessed in six *HER2*-negative GC cell lines, with NUGC4 and SNU719 showing the highest sensitivity (Fig. 1C and S1C, D). FISH confirmed *EGFR*-high CN in NUGC4 and SNU719, whereas SGC7901 and MGC803 displayed *EGFR*-low CN (Fig. 1D and S1E). To further evaluate pyrotinib’s effects on apoptosis and tumor growth in GC models with different EGFR CN status, Annexin V/PI staining, western blot and subcutaneous xenogarft experiments were performed. Flow cytometry analysis demonstrated that pyrotinib dose-dependently induced apoptosis in *EGFR*-high CN GC cells, whereas no significant effect was observed in *EGFR*-low CN counterparts (Fig. 1E and S1F). Western blot analysis revealed dose-dependent upregulation of cleaved caspase-9 in *EGFR*-high CN GC cells treated with pyrotinib (0–2 µM), with no significant changes in *EGFR*-low CN cells (Fig. 1F). In vivo, pyrotinib induced significant tumor regression in *EGFR*-high CN GC derived xenografts, whereas no inhibition was observed in *EGFR*‐low CN models (Fig. 1G and S1G). Immunohistochemical analysis corroborated these results, showing reduced Ki-67 proliferation indices and p-EGFR levels in responsive tumors (Fig. 1H). Additionally, TUNEL assays confirmed enhanced apoptosis in pyrotinib-treated NUGC4 and SNU719 xenografts (Fig. 1I and S1H).

To further elucidate pyrotinib’s mechanism of activity and identified the potential targets of pyrotinib, we analyzed phosphorylation statuses of EGFR, HER2 and HER4 – the established therapeutic targets of this pan-HER inhibitor across seven GC cell lines following pyrotinib treatment. As shown in Fig. 1J, pyrotinib-sensitive cells (SNU719, NUGC4; *EGFR*-high CN and *HER2/HER4*-low CN) exhibited significant suppression of p-EGFR and its downstream p-AKT, whereas p-HER2/p-HER4 suppression was insignificant in AGS, SNU601, MGC803, SGC7901 (*EGFR/HER2/HER*4-low CN). This differential efficacy stems from intrinsically low basal phosphorylation of HER2/HER4 in low-CN cells, a key determinant of pyrotinib’s cell type-dependent activity.

Functional validation using si*RNA* knockdown revealed that EGFR silencing, but not *HER2* or *HER4* knockdown, rescued pyrotinib-induced apoptosis, as confirmed by Annexin V/PI staining assays (Fig. 1K and S1I, J). Similarly, sh*EGFR*-transfected GC cells displayed reduced pyrotinib sensitivity compared to sh*NC* cells (Fig. 1L and S1K).

### Pyrotinib synergizes with oxaliplatin to suppress *EGFR*-high CN gastric cancer growth

**Fig. 2:**
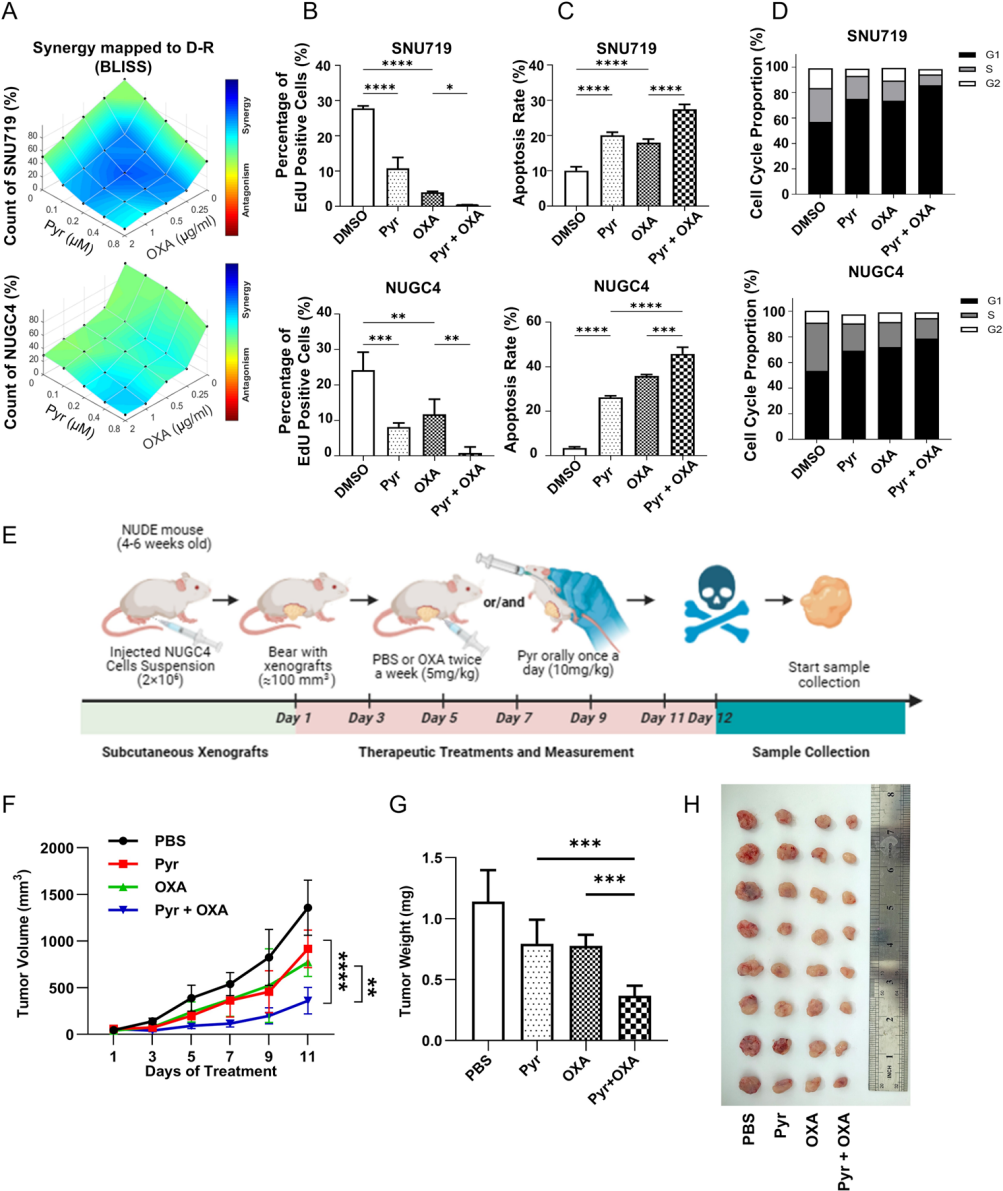
Pyrotinib combined with oxaliplatin exerts synergistic effects in EGFR- high CN gastric cancer. **A**. Drug synergy maps of SNU719 and NUGC4 cells were plotted by Combenefit software (V2.021). Bliss synergy scores were calculated for oxaliplatin (OXA) and Pyr after 48 h. One representative experiment out of 3 independent experiments is shown. **B**. Quantitative analysis of EdU positive in SNU719 and NUGC4 cells treated with Pyr (1 µM) or/and OXA (1 µg/ml) for 24 h. **C**. Quantitative analysis of Annexin V-FITC/PI staining after the indicated treatment. **D**. Quantitative analysis of cell cycle distribution in SNU719 and NUGC4 cells after the indicated treatment. **E**. Schematic diagram of the protocol for the animal model experiments. **F**. Tumor volume curves were plotted every 2 days after indicated treatment (*n* = 8). **G**-**H**. Tumor weight (**G**) and maximum diameter (**H**) were recorded after excision from xenograft models. Significance was determined by Paired t-test (**F**) and (**B**, **C**, **G**) One-way ANOVA. *p < 0.05; **p < 0.01; ***p < 0.001

Given the clinical application of oxaliplatin-based chemotherapy in advanced GC, we systematically evaluated the therapeutic synergy between pyrotinib and oxaliplatin. Dose-response matrix analysis revealed synergistic interactions in *EGFR*-high CN GC cell lines (Fig. 2A). EdU incorporation assays confirmed superior anti-proliferative activity of the drug combination, compared to oxaliplatin or pyrotinib treatment alone (Fig. 2B and S2A). Apoptosis assays demonstrated increased apoptotic populations in combination-treated SNU719 and NUGC4 cells compared to monotherapies (Fig. 2C and S2B). To investigate how the oxaliplatin-pyrotinib combination inhibits proliferation, we performed cell cycle analysis. The combination significantly increased G0/G1 phase accumulation compared to monotherapies, indicating cell cycle arrest-mediated growth inhibition (Fig. 2D and S2C). This arrest blocks DNA replication to inhibit proliferation and sensitizes cells to OXA-induced DNA damage, underpinning Pyr-OXA therapeutic synergy. Based on the observed in vitro synergy between pyrotinib and oxaliplatin, we established NUGC4 xenograft models to evaluate their individual and combined antitumor efficacy. The combination therapy demonstrated superior tumor growth suppression compared to either agent alone, with maximal efficacy observed at treatment endpoint (Fig. 2E-H). Notably, no significant body weight loss was observed as a toxicity metric (Fig. S2D).These results altogether demonstrated that the pyrotinib-oxaliplatin combination exerts enhanced anti-tumor effects in vitro and in vivo.

### Pyrotinib induces ER stress-mediated apoptosis through the PERK/eIF2α/ATF4/CHOP axis in *EGFR*-high CN gastric cancer

EGFR, a critical receptor tyrosine kinase, that regulates multiple signaling pathway such as PI3K/AKT or /MAPK/ERK pathway [31]. However, pyrotinib promoted apoptosis in presence of MK2206 (AKT inhibitor) and ASN007 (ERK inhibitor) (Fig. S3A), indicating it depends on a non-canonical EGFR kinase pathway.

**Fig. 3:**
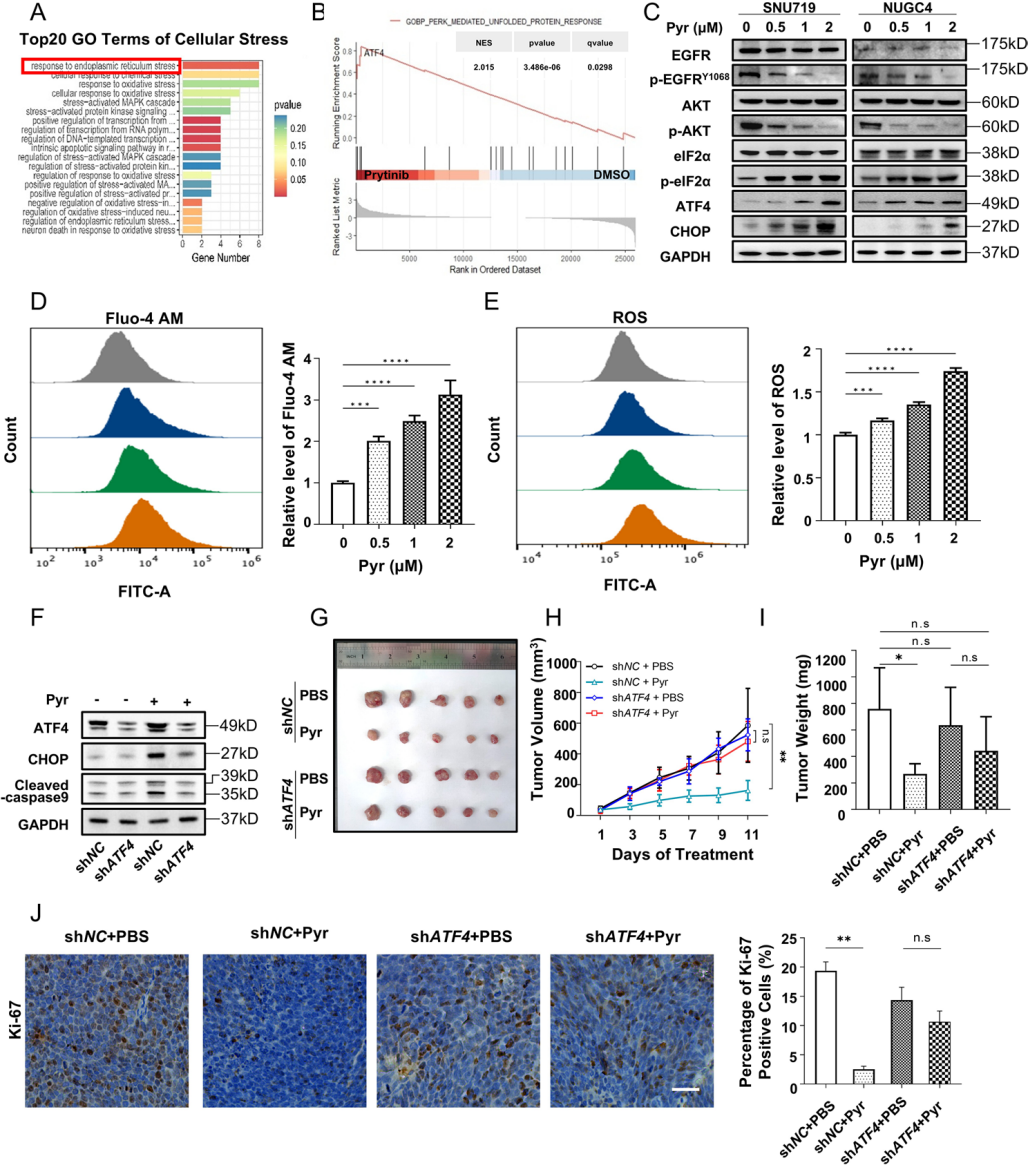
PERK/eIF2α/ATF4/CHOP mediated endoplasmic reticulum stress is involved in regulation of pyrotinib associated injuring in EGFR-high CN GC. **A**. Stress-related pathways gene ontology (GO) enrichment analysis highlighted the activation of ER stress and UPR in SNU719 cells treated with pyrotinib (1 µM) compared to DMSO.**B**. GSEA plot identified significant enrichment of unfolded protein response (UPR) pathway with prominent ATF4 expression in NUGC4 derived xenograft treated with pyrotinib (1 µM) compared to PBS. **C**. Lysates of SNU719 and NUGC4 cells treated with pyrotinib for 48 h were blotted with the indicated antibodies. p-AKT^S473^ and p-EGFRY^1068^ was used to monitor the effect of pyrotinib. **D**. Ca^2+^ of SNU719 cells were monitored using Fluo-4 AM by flow cytometry after treatment of increasing doses of pyrotinib for 48 h. **E**. The cellular ROS levels were also analyzed by flow cytometry. **F**. Western blot analysis showed the expression levels of ATF4, CHOP, and cleaved caspase-9 in SNU719 cells transfected with sh*NC* or sh*ATF4*, and treated with or without pyrotinib (1 µM). **G**-**J**. Nude mice were injected subcutaneously in the right groin with sh*NC* or sh*ATF4* SNU719 cells and treated with PBS or pyrotinib (10 mg/kg) per day. After 11 days treatment, xenografts were harvested for experiments. (**H**) Tumor volume was recorded every 2 days. (**I**) tumor weight of excised xenografts was determined after sacrificed. (**J**) Representative IHC imaging (left panel) and quantitative analysis (right panel) of Ki-67 stained tumor from xenograft models. Bar, 100 μm. Significance was determined by (**H**) Paired t-test and (**D**, **E**, **I**, **J**) One-way ANOVA. *p < 0.05; **p < 0.01; ***p < 0.001

To explore the molecular mechanisms underlying pyrotinib’s anti-tumor activity, we performed transcriptomic profiling of pyrotinib-treated xenograft tumors in vivo. Gene ontology (GO) analysis revealed significant enrichment of ER stress-related pathways in pyrotinib-treated versus PBS in SNU719 cells (Fig. 3A). Additionally, gene set enrichment analysis (GSEA) in NUGC4-derived xenografts treated with pyrotinib or not demonstrated upregulation of PERK-mediated unfolded protein response (UPR) pathways (Fig. 3B). Consistent with these findings, immunoblotting confirmed dose-dependent upregulation of ER stress-associated markers (p-eIF2α, ATF4, CHOP) in SNU719 and NUGC4 cells (Fig. 3C). ER stress is mechanistically linked to calcium homeostasis dysregulation and reactive oxygen species (ROS) generation [32]. Consistent with this, pyrotinib treatment induced dose-dependent elevations in intracellular calcium levels of Ca²⁺ (Fig. 3D) and ROS (Fig. 3E).

Given the central role of ATF4 in PERK-mediated ER stress activation and distinct changes in RNA-seq (Fig. S3B), we genetically knocked down *ATF4* using sh*RNA* to assess its functional contribution. At the same time, ATF4/CHOP axis was responsible for apoptosis and caspase activation in cancer [33]. *ATF4* knockdown significantly attenuated pyrotinib-induced C/EBP homologous protein (CHOP) upregulation and caspase activation (cleaved caspase-9) (Fig. 3F). Furthermore, in SNU719 xenograft models, *ATF4* silencing partially reversed pyrotinib’s therapeutic efficacy (Fig. 3G), manifesting as attenuated tumor growth suppression (reduced volume and weight) (Fig. 3H, I) and elevation of Ki-67 proliferation indices (Fig. 3J).

### eIF2α phosphatase inhibition enhances pyrotinib’s efficacy

**Fig. 4:**
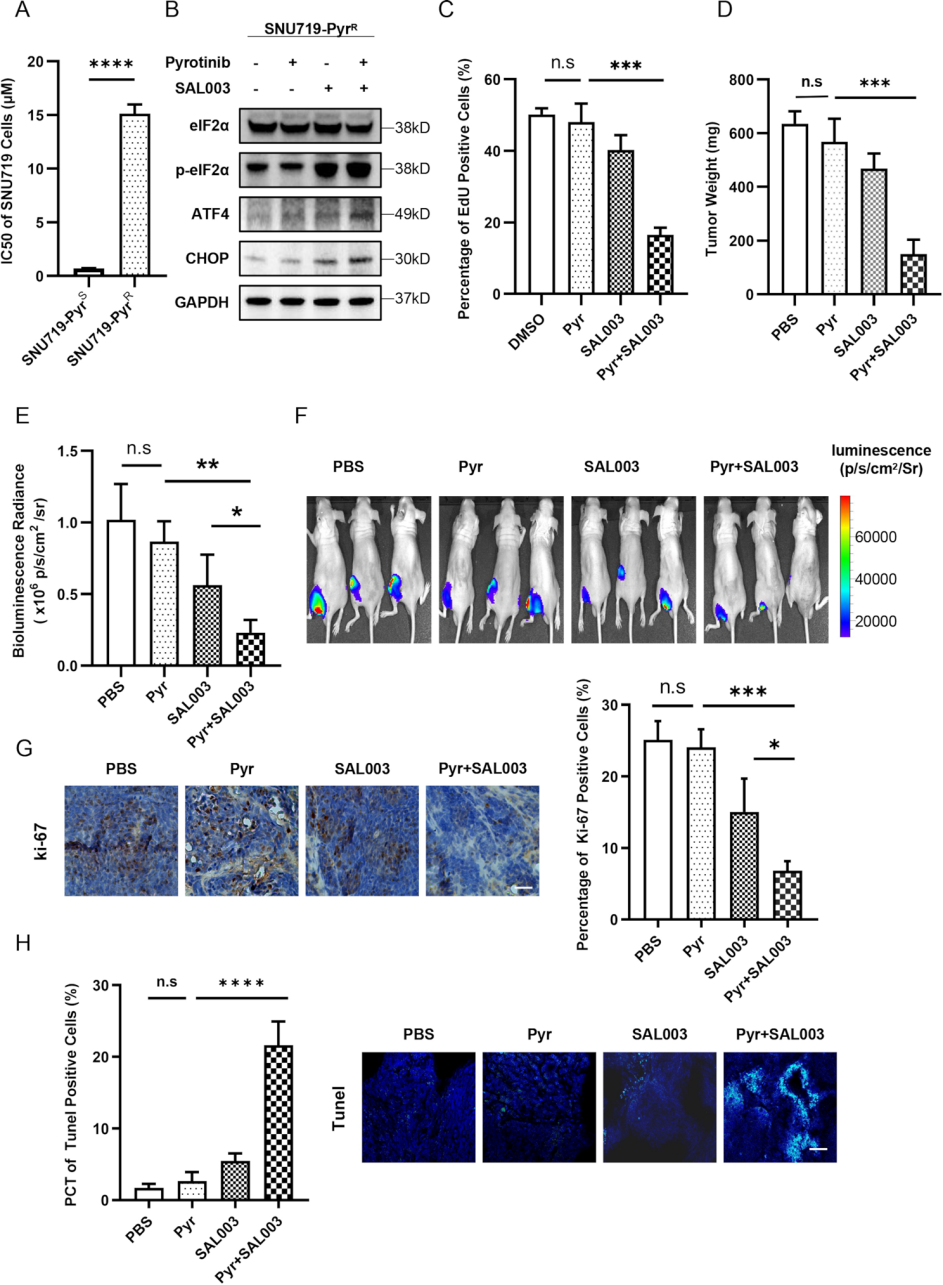
Elevated p-eIF2α level can reverse acquired resistance to pyrotinib. **A**. IC50 values of Pyr in parental SNU719 (SNU719-Pyr^S^) and pyrotinib resistant (SNU719-Pyr^R^) cells. **B**. WB analysis of protein extracted from SNU719-Pyr^R^ cells treated with pyrotinib (1 µM), SAL003 (10 µM), or their combination for 48 h. **C**. EdU assays demonstrated the inhibitory effects of pyrotinib (1 µM), SAL003 (10 µM) or their combination on the proliferation of SNU719-Pyr^R^ cells. **D**-**F**. A total of 20 nude mice were subcutaneously implanted with SNU719-Pyr^R^ cells into the groin and randomly assigned to 4 treatment groups (*n* = 5/group). After 7 days post-injection, mice were orally administered with pyrotinib (10 mg/kg daily) or PBS, intratumorally injected with SAL003 (10 µM), or combination. (**D**) Tumor weight and (**E**) bioluminescent intensity were quantified at the day 21. (**F**) Representative bioluminescent images of tumors. **G**-**H**. Immunohistochemical staining for Ki-67 (**G**) and TUNEL assay (**h**) illustrated representative microscopic images of tumor suppression effects induced by pyrotinib and SAL003 in SNU719-Pyr^R^ induced xenograft models. Bars, 200 μm. (**A**, **C**, **D**, **E**, **G**, **H**) Significance was determined by One-way ANOVA. *p < 0.05; **p < 0.01; ***p < 0.001

To investigate the effect of PERK/eIF2α/ATF4/CHOP axis in pyrotinib-mediated cell injury, we established an acquired pyrotinib-resistant cell line (SNU719-Pyr^R^) from parental SNU719 cells, which exhibited a significantly higher IC50 compared to the parental line (Fig. 4A and S4A). This model replicates clinical acquired resistance to pyrotinib, thus establishing a platform to identify and overcome such resistance through mechanism-guided therapeutic strategies. Contrast to pyrotinib-sensitive SNU719 cells (SNU719-Pyr^S^), pyrotinib failed to suppress AKT phosphorylation or activate the PERK/eIF2α/ATF4/CHOP pathway in SNU719-Pyr^R^ cells; however, co-treatment with pyrotinib and SAL003 (an eIF2α phosphatase inhibitor) restored these signaling effects in SNU719-Pyr^R^ cells (Fig. 4B). Consistent with these findings, the combination of SAL003 and pyrotinib demonstrated superior anti-proliferative activity to pyrotinib alone in SNU719-Pyr^R^ cells, as evidenced by EdU assays (Fig. 4C and S4B). To validate these observations in vivo, we evaluated the effects of SAL003 and pyrotinib on SNU719-Pyr^R^-derived xenografts. Tumor weights (Fig. 4D and S4C) and bioluminescence intensity (Fig. 4E, F) revealed that monotherapy with either SAL003 or pyrotinib induced partial tumor suppression, whereas combination therapy achieved significantly enhanced efficacy. Immunohistochemical analysis further confirmed reduced Ki-67 proliferation indices in tumors treated with the combination (Fig. 4G). TUNEL assays also showed elevated apoptosis rate in tumors administered with the combination group (Fig. 4H).

To elucidate the molecular basis of acquired resistance in SNU719-Pyr^R^ cells, we performed RNA sequencing and differential gene expression analysis. *ATF4* downregulation was consistently observed (Fig. S4D), aligning with the suppression of PERK/ATF4/CHOP axis in resistant cells. KEGG pathway enrichment further revealed compensatory upregulation of pro-survival pathways, including DNA replication, mismatch repair, cell cycle, metabolic reprogramming (specifically nitrogen metabolism), and PPAR signaling et al., which conferring evasion of pyrotinib-induced ER stress (Fig. S4E).This model replicates clinical acquired resistance to pyrotinib, thus establishing a platform to identify and overcome such resistance through mechanism-guided therapeutic strategies.

### Pyrotinib drives EGFR translocated to ER and interacted with GRP78

**Fig. 5:**
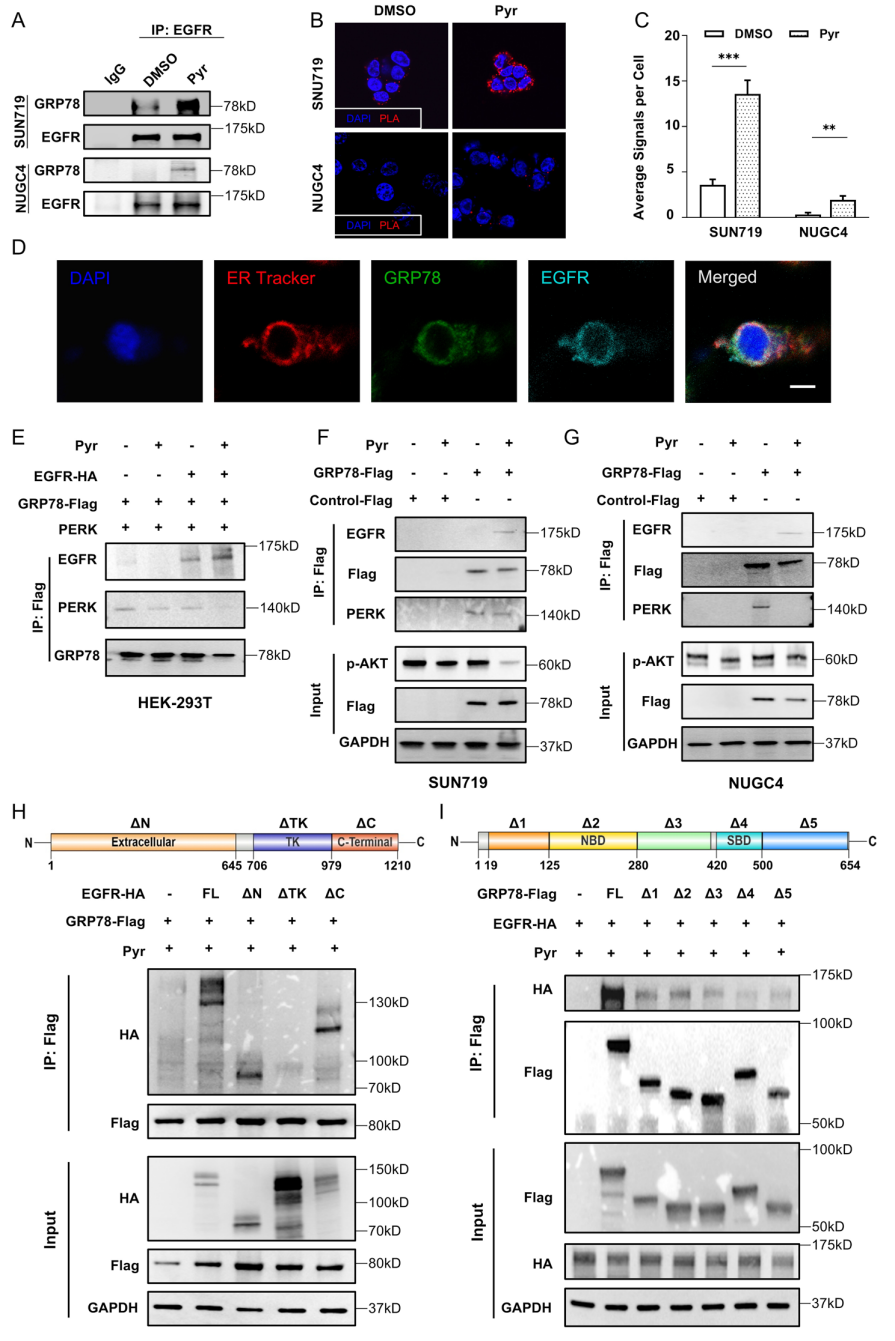
Pyrotinib drives EGFR translocated to ER and interacted with GRP78. **A**. Lysates from SNU719 and NUGC4 cells treated with DMSO or Pyr (1 µM) were precipitated with EGFR antibodies by immunoprecipitation. The precipitated proteins were assayed by western blot. **B**-**C**. Representative images (**B**) and quantification (**C**) of the proximity ligation assay (PLA) detecting EGFR-GRP78 interactions in SNU719 and NUGC4 cells treated with DMSO or Pyr (1µM). Red dots indicate PLA signals. Scale bar, 40 μm. Significance between groups was determined by One-way ANOVA. *p < 0.05; **p < 0.01; ***p < 0.001. **D**. Immunofluorescence analysis of ER-tracker (red), GRP78 (green), and EGFR (cyan) in SNU719 cells treated with Pyr (1µM). Scale bar, 50 μm. **E**. HEK293T cells were transfected with the indicated plasmid, treated with or without Pyr (1µM), followed by immunoprecipitation with anti-flag antibody, and eventually assayed by WB analysis with the indicated antibodies. **F**-**G**. SNU719 (**F**) and NUGC4 (**G**) cells were transfected with flag-GRP78 and treated with or without Pyr (1 µM) for 48 h. Cell lysates were subjected to immunoprecipitation using an anti-Flag antibody, followed by western blot analysis with the indicated antibodies. **H**. HEK293T cells were co-transfected with GRP78-flag and EGFR-HA (FL: full length, or its truncation mutants) plasmids for 48 h, followed by co-immunoprecipitation (CO-IP) assay. **I**. HEK293T cells were co-transfected with EGFR-HA and GRP78-FLAG (FL or its truncation mutants) plasmids for 48 h and treated with Pyr (1 µM) for 48 h, followed by CO-IP assay

The unfolded protein response (UPR), mediated by endoplasmic reticulum stress, is a critical mechanism for maintaining proteostasis. Under physiological conditions, GRP78/BIP – a key ER stress sensor – remains inactive in phosphorylation state through its association with the PERK. We hypothesized that pyrotinib-induced EGFR dephosphorylation might enhance EGFR-GRP78 interaction. Co-IP assays (Fig. 5A) and proximity ligation assays (PLA) (Fig. 5B, C) revealed enhanced EGFR-GRP78 complex formation following pyrotinib treatment. Multiplex fluorescence imaging showed pyrotinib treatment induced EGFR translocated from cell membrane to ER (Fig. S5A), and co-localized with GRP78 (Fig. 5D). Notably, pyrotinib suppressed GRP78-PERK binding while promoting GRP78-EGFR interaction (Fig. 5E-G). To map the interaction domains, we generated EGFR truncation mutants lacking specific regions in 293T cells. EGFR deletion mutants lacking the tyrosine kinase domain, the target domain of pan-HER inhibitors, failed to interact with GRP78, suggesting this domain mediates pyrotinib-dependent complex formation (Fig. 5H). Meanwhile, deletion of the C-terminal domain (Δ420–500 and Δ500–654) abolished GRP78-EGFR binding, whereas other truncations retained this interaction (Fig. 5I). Three-dimensional models showed the pyrotinib-induced interaction of EGFR and GRP78 (Fig. S5B). Based on these findings, we proposed that pyrotinib disrupts GRP78-PERK binding and promotes GRP78-EGFR interaction, thereby facilitating PERK/ATF4/CHOP axis activation.

### Pyrotinib accelerated the ubiquitination of GRP78 via blocking Thr62 phosphorylation

**Fig. 6:**
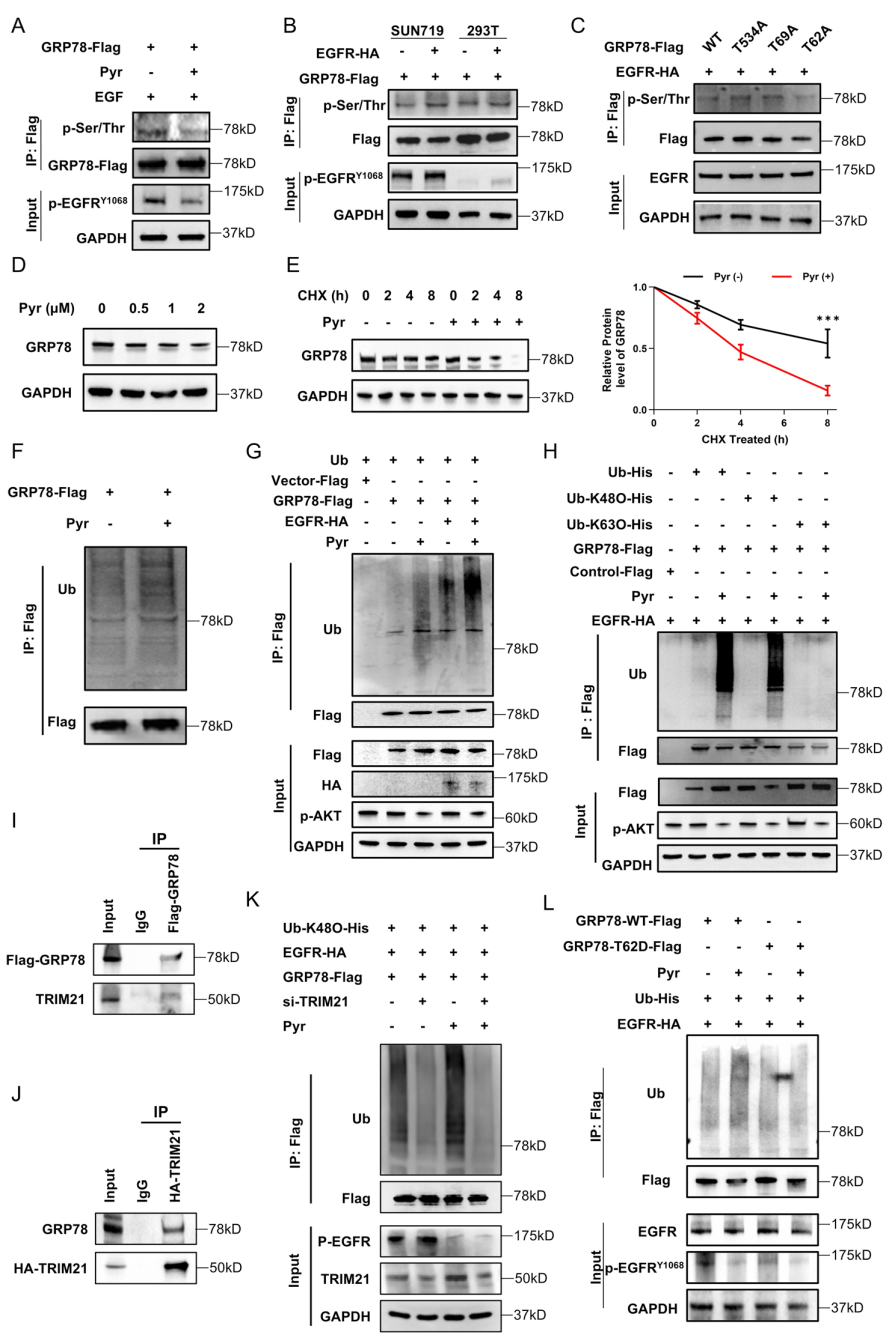
Pyrotinib accelerated the ubiquitination of GRP78 via blocking Thr62 phosphorylation. **A**. CO-IP assays were performed to assess GRP78 phosphorylation in SNU719 cells treated with EGF stimulation (100 ng/mL) for 5 min, followed with or without treatment of Pyr for 24 h. The lysates were incubated with indicated antibodies. **B**. CO-IP assays were performed to detect the phosphorylation of GRP78 in EGFR-overexpressing or/and GRP78-overexpressing SNU719 cells and HEK-293T cells after stimulating with EGF (100 ng/mL) for 5 min. **C**. Mutational analysis of GRP78 residues (T62A, T69A, T534A) identified critical phosphorylation sites mediating Pyr-induced GRP78 dephosphorylation. **D**. Western blot analysis revealed dose-dependent reductions in GRP78 protein levels after 24 h treatment with increasing Pyr concentrations in SNU719 cells. **E**. Cycloheximide (CHX, 100 µg/mL) chase experiments demonstrated accelerated GRP78 degradation in Pyr (1 µM, 24 h) treated versus untreated SNU719 cells. **F**. SNU719 cells co-transfected with Ub-HA and GRP78-Flag plasmids were treated with Pyr (1 µM) and MG132 (10 µM). Immunoblotting confirmed enhanced GRP78 ubiquitination under proteasomal inhibition. **G**. HEK293T cells were co-transfected with the indicated plasmids for 48 h and then treated with MG132 (10 µM). The polyubiquitination levels of GRP78 protein were analyzed. **H**. HEK293T cells expressing GRP78-Flag with WT ubiquitin or mutant (Ub-K48O, Ub-K63O) plasmids were treated with Pyr (1 µM, 24 h). Ubiquitination patterns confirmed K48-linked polyubiquitination as the dominant degradation signal. **I**. Immunoprecipitation was performed in SNU719 lysates transfected with flag-GRP78 using Flag antibodies, and the precipitants were measured by western blotting with the indicated antibodies. **J**. Immunoprecipitation was performed in SNU719 lysates transfected with HA-TRIM21 using HA antibodies, and the precipitants were measured by western blotting with the indicated antibodies. **K**. SNU719 cells co-transfected with indicated plasmids and/or si*TRIM21*, then treated with or without Pyr (1 µM). WB analysis showed reduced polyubiquitination of the si*TRIM21* group compared to control. **L**. HEK293T cells co-transfected with indicated plasmids and treated with MG132 (10 µM) showed reduced polyubiquitination of the T62D mutant compared to WT GRP78 upon Pyr (1 µM, 24 h) stimulation. Significance between groups was determined by (**E**, **I**) Paired t-test. *p < 0.05; **p < 0.01; ***p < 0.001

Given the observed EGFR-GRP78 interaction and pyrotinib-mediated EGFR phosphorylation inhibition, we next investigated whether pyrotinib similarly modulates GRP78 phosphorylation status. Co-IP assay revealed decreased phosphorylated GRP78 levels in pyrotinib-treated SNU719 cells, whereas EGFR-overexpressing cells exhibited phosphorylation enhancement (Fig. 6A, B). Through PhosphoSite Plus database screening of potential phosphorylation sites (Thr62, Thr69, Thr534), Thr62 of GRP78 was identified as the primary residue activated by EGFR (Fig. 6C).

Notably, pyrotinib selectively downregulated GRP78 protein (Fig. 6D and S6A) but not mRNA levels (Fig. S6B) in *EGFR*-high CN cells, indicating post-translational regulation. Cycloheximide (CHX) chase assays revealed that EGFR inhibition shortened GRP78 half-life (Fig. 6E), suggesting that inactivation of the EGFR pathway appears to destabilize GRP78 protein. As ubiquitination and lysosomal degradation are key proteolytic pathways [26, 34], we assessed their contributions. Proteasome inhibition by MG132 fully restored GRP78 levels in pyrotinib-treated cells, whereas lysosomal blockade (leupeptin) had no effect (Fig. S6C). Consistently, pyrotinib increased poly-ubiquitinated GRP78 (Fig. 6F). As expected, GRP78 ubiquitination was upregulated in EGFR-overexpression cells than that of mock cells (Fig. 6G), indicating that the EGFR signaling pathway is involved in the proteasomal degradation of GRP78. To define ubiquitin chain specificity, we utilized lysine-restricted ubiquitin mutants (K48O: retains Lys48 only; K63O: retains Lys63 only). K48O, but not K63O, increased GRP78 ubiquitination (Fig. 6H), demonstrating K48-linked ubiquitination drives pyrotinib-induced degradation.

To identify the E3 ligase responsible for K48-linked GRP78 ubiquitination, we focused on TRIM21, implicated in GRP78 degradation in non-small cell lung cancer [18]. Co-IP assays in gastric cancer cells confirmed the GRP78-TRIM21 complex (Fig. 6I, J). CHX chase assays revealed that ectopic TRIM21 overexpression markedly accelerated GRP78 degradation in SNU719 cells versus vector controls (Fig. S6D), indicating TRIM21 promotes GRP78 degradation. Finally, si*RNA*-mediated *TRIM21* knockdown in HEK293T cells attenuated pyrotinib-induced K48-linked polyubiquitination of GRP78 with or without pyrotinib (Fig. 6K), confirming TRIM21’s essential role in this specific modification.

As phosphorylation often regulates ubiquitin ligase recognition and previous studies showed phosphorylation of GRP78 inhibit its own ubiquitination, we generated a phospho-deficient GRP78 mutant (T62A) and found T62A mutant accelerated the degradation of GRP78 (Fig. S6E). Next, we also generated T62D phosphorylation-mimic mutant and found it was resistant to pyrotinib-induced ubiquitination (Fig. 6L).

Collectively, these findings demonstrate that pyrotinib promotes GRP78 degradation by suppressing EGFR-mediated phosphorylation at Thr62. This dephosphorylation specifically facilitates TRIM21 recruitment, thereby triggering K48-linked polyubiquitination and ultimately proteasomal degradation of GRP78. Critically, Thr62 phosphorylation status governs TRIM21-dependent degradation of GRP78.

### Pyrotinib enhances oxaliplatin chemosensitivity through GRP78 inhibition

**Fig. 7:**
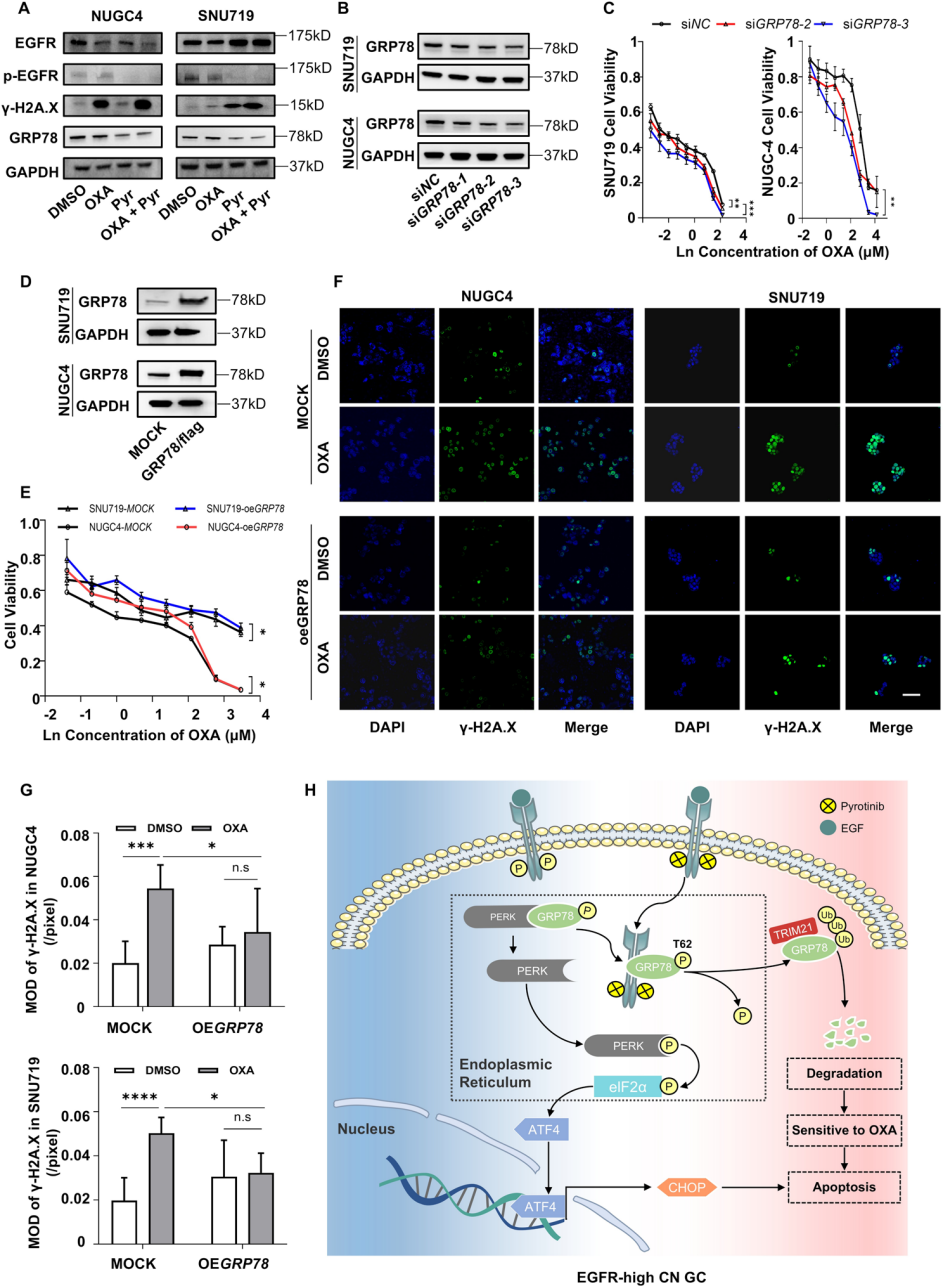
Pyrotinib enhances oxaliplatin chemosensitivity through GRP78 inhibition. **A**. Western blot analysis revealed differential expression of DNA double-strand break repair proteins (γ-H2A.X) and GRP78 in NUGC4 and SNU719 cells treated with DMSO, Pyr (1 µM), or/and OXA (2.5 µM) for 24 h. **B**. The expression of GRP78 protein in SNU719 and NUGC4 cells transfected with si*NC* or si*GRP78*. **C**. CCK-8 cytotoxicity assays demonstrated enhanced OXA sensitivity in GRP78 silenced SNU719 and NUGC4 cells. **D**. The expression of GRP78 protein in SNU719 and NUGC4 cells transfected with MOCK or OE*GRP78* plasmid. **E**. GRP78 overexpressing GC cells exhibited OXA resistance in CCK-8 assays, showing higher viability than control cells. **F**-**G**. Immunofluorescence staining (**F**) and analysis (**g**) showed that after stimulation by OXA (2.5 µM) for 24 h, the γ-H2A.X foci in SNU719 and NUGC4 cell nucleus distinguished in MOCK and OE*GRP78* groups. Blue background indicates DAPI, red dots indicate γ-H2A.X foci. Bars, 40 μm. **H**. Schematic diagram display that Pyr promotes EGFR-GRP78 complex formation in the ER lumen, subsequently activating the PERK/eIF2α/ATF4/CHOP signaling pathway, and finally resulted in the tumor apoptosis in EGFR-high CN GC. Besides, Pyr could sensitize GC cells to OXA by inhibiting GRP78 phosphorylation at T62 which results in GRP78 ubiquitination. Significance between groups was determined by (**C**, **E**) Paired t-test and (**G**) One-way ANOVA. *p < 0.05; **p < 0.01; ***p < 0.001

Previous studies have demonstrated that EGFR-targeted inhibitors (cetuximab) could impair DNA double-strand break (DSB) repair mechanisms to increase radio-sensitivity [15]. Building on this evidence, we hypothesized that pyrotinib-induced GRP78 silencing would suppress oxaliplatin-triggered DNA damage response through DSB repair pathway modulation. Western blot analysis revealed that pyrotinib significantly attenuated oxaliplatin-induced expression of γ-H2A.X, a key DSB repair biomarker, in GC cells (Fig. 7A). Pyrotinib further increased the expression of oxaliplatin-induced ER stress-associated proteins in vitro (Fig. S7A). To determine whether ER stress is essential for the synergistic pro-apoptotic effect of pyrotinib and oxaliplatin, we performed rescue experiments using the ER stress inhibitor 4-PBA (10 µg/mL). Notably, flow cytometry analysis revealed that co-treatment with pyrotinib and oxaliplatin significantly increased apoptosis in SNU719 cells with or without ER stress inhibition (Fig. S7B, C). Consistent with this, WB analysis confirmed sustained cleavage caspase-9 in the combination group despite 4-PBA co-administration (Fig. S7D). These results demonstrate that the synergistic pro-apoptotic effect is not exclusively dependent on ER stress pathways, but is primarily driven by GRP78 ubiquitination-mediated DNA repair impairment.

Functional validation demonstrated that GRP78 knockdown substantially enhanced cellular sensitivity to oxaliplatin (Fig. 7B, C), whereas GRP78 overexpression conferred chemoresistance (Fig. 7D, E). Notably, γ-H2A.X foci formation showed significant elevation in oxaliplatin-treated control groups but remained unchanged in GRP78-overexpressing cells (Fig. 7F, G). Taken together, these findings demonstrated that pyrotinib could enhance oxaliplatin chemosensitivity through GRP78 downregulation mediated impairment of DNA repair mechanisms (Fig. 7H).

## Discussion

The HER family represents one of the most extensively targeted receptor families in oncology due to its frequent dysregulation across malignancies. Previous studies have established that afatinib sensitivity correlates with *EGFR/ERBB2* co-amplification in gastric cancer [35], highlighting the prospective therapeutic potential of pan-HER inhibitors in clinical practice. Pyrotinib, an oral pan-HER inhibitor, is widely used in patients with *HER2*-mutated non-small-cell lung cancer and *HER2*-amplicated breast cancer [36, 37] and displays clinical activity in *HER2*-positive or *HER3*-mutated gastric cancer [38, 39].

In this study, we systematically screened and identified pyrotinib as a potent inhibitor selectively suppressing *EGFR*-high CN GC cells both in vitro and in vivo. Previous studies demonstrated anti-tumor effect of pyrotinib in HER2(+) GC [40]. Our findings reveal a strong correlation between pyrotinib response and *EGFR* high CN, independent of *HER2* or *HER4* status. This aligns with prior observations that cetuximab, an anti-EGFR monoclonal antibody, benefits a subset of GC patients with *EGFR* amplification [8]. Notably, pyrotinib has been reported to induce apoptosis in colon cancer and oral squamous cell carcinoma by targeting EGFR [41, 42], underscoring its broader therapeutic relevance. While lapatinib, another pan-HER inhibitor, failed to improve overall survival in HER2-amplified gastroesophageal adenocarcinoma when combined with CapeOx, subgroup analyses suggested prolonged survival in Asian and younger populations [43]. Similarly, another pan-HER inhibitor afatinib has shown a good response in EGFR-mutated GC in clinical practice [44]. However, EPHA2, which is participates in crosstalk with other membrane receptors and impinged on downstream RAS/PI3K/AKT and RAS/MAPK signaling pathways, might be involved in the mechanism of acquired resistance to EGFR inhbitors in treating GC [12]. Together with our study, we highlight pan-HER inhibitors may be a viable strategy for GC subsets characterized by EGFR hyperactivity. Our data demonstrate that pyrotinib synergizes with oxaliplatin to induce ER-stress and activate the PERK-ATF4-CHOP apoptotic cascade, a key pathway that transcriptionally represses Bcl-2 while upregulating pro-apoptotic BIM and cleaved caspase-3 [45]. This CHOP-mediated process eliminates stress-damaged gastric cancer cells through intrinsic apoptosis, thereby synergizing with OXA-driven DNA damage to achieve tumor suppression [46].

*EGFR* high CN is associated with chromosomal instability and poor prognosis in advanced GC [47]. Current therapeutic paradigms increasingly favor combining anti-EGFR agents with chemotherapy to overcome resistance while minimizing toxicity. Prior studies have demonstrated that underglycosylated EGFR forms stable complexes with GRP78 [48]. Notably, pyrotinib has been shown to enhance radiosensitivity and chemosensitivity in *HER2*-positive GC [49]. Here, we demonstrate that pyrotinib synergizes with oxaliplatin to augment apoptosis in *EGFR*-high CN GC. Mechanistically, this synergy arises from dual effects: EGFR inhibition induces ER stress-mediated apoptosis via the PERK/ATF4/CHOP axis, while GRP78 downregulation impairs DNA damage repair by suppressing BRCA1-mediated homologous recombination repair [50], thereby potentiating oxaliplatin-induced DSBs [51]. Prior studies indicate that ER stress compromises DSB repair, increasing chemosensitivity [52], while EGFR blockade induces ER stress in cancer cells [13, 53]. Our work bridges these concepts by establishing pyrotinib as a dual modulator of ER stress and DNA repair pathways in *EGFR*-driven GC.

GRP78, a central ER chaperone, undergoes phosphorylation-dependent regulation that influences cancer progression [21]. Structurally, GRP78 harbors ATPase and autophosphorylation activities critical for its function in the endoplasmic reticulum [54]. GRP78 phosphorylation, a process linked to spatial reorganization during sperm epididymal maturation, is markedly elevated in cancer cells compared to normal cells [17], underscoring its critical role in cancer progression. Our study reveals that pyrotinib suppresses GRP78 phosphorylation at Thr62, which may explain a novel mechanism for killing cancer cells. Furthermore, crosstalk between ubiquitination and phosphorylation has emerged as a key regulatory axis in cancer [55]. For instance, SCNN1B and GSC1 modulate GC progression by regulating GRP78 ubiquitination [56, 57]. Besides, X-linked inhibitor of apoptosis-associated factor-1 interacts with and destabilizes ER stress sensor GRP78 and increased cell sensitivity to ER stress [58]. Actually, our findings showed that inhibition of EGFR activity by pyrotinib blocks GRP78 phosphorylation, subsequently improves GRP78 ubiquitination and proteasomal degradation, and finally enhance apoptosis induced by oxaliplatin stress in *EGFR*-high CN GC cells.

In summary, our study not only reveals for the first time the significant therapeutic potential of pyrotinib combined with oxaliplatin in targeting *EGFR*-high CN GC, but also reveals the functional role of the EGFR/GRP78/ATF4/CHOP axis in anti-EGFR therapeutic strategies. These findings collectively propose a novel treatment regimen – pyrotinib plus oxaliplatin – as a promising option for posterior-line therapy in GC with *EGFR*-high CN.

## Conclusions

Our findings demonstrate that pyrotinib selectively targets GC with *EGFR*-high CN by mediating dual mechanisms: (1) facilitating EGFR-GRP78 complex formation in the endoplasmic reticulum to activate the PERK/ATF4/CHOP axis, thereby triggering ER stress-mediated apoptosis, and (2) suppressing GRP78 phosphorylation at Thr62, which promotes K48-linked ubiquitination and proteasomal degradation, impairing DNA repair and synergizing with oxaliplatin to amplify chemosensitivity. This study uncovers the pivotal role of the EGFR/GRP78 signaling axis and establishes pyrotinib-oxaliplatin combination therapy as a novel strategy for GC with *EGFR*-high CN.

## Electronic supplementary material

Below is the link to the electronic supplementary material.

Supplementary Material 1

Supplementary Material 2

## Data Availability

No datasets were generated or analysed during the current study.

## Abbreviations

GC: Gastric cancer
EGFR: Epidermal growth factor receptor
ER: Endoplasmic reticulum
GRP78: Glucose-regulated protein 78
PERK: Pancreatic ER kinase (PKR)-like ER kinase
CHOP: The C/EBP homologous protein
CN: Copy number
DSB: Double-strand break
FISH: Fluorescence in situ hybridization
