# Supplementary material for: Pyrotinib targeted EGFR/GRP78 mediated cell apoptosis in high EGFR gene copy number gastric cancer
Source: J Exp Clin Cancer Res. 2025 Aug 19;44:245. doi: 10.1186/s13046-025-03485-6 (PMC12363034; doi:10.1186/s13046-025-03485-6)
Supplement: Supplementary file 1 — Supplementary Material 1 [file 13046_2025_3485_MOESM1_ESM.docx]

**Supplementary Table 1.** The sequences of sh*RNA*.

|  | 5’-3’ |
| --- | --- |
| sh*NC* | TTCTCCGAACGTGTCACGT |
| sh*EGFR-1* | CGCAAAGTGTGTAACGGAATA |
| sh*EGFR-2* | CATCAGTGGCGATCTCCACAT |
| sh*EGFR-3* | CTGGATCCACAGGAACTGGAT |
| sh*ATF4* | CCACTCCAGATCATTCCTTTA |

**Supplementary Table 2.** The sequences of si*RNA*.

|  | 5’-3’ | 3’-5’ |
| --- | --- | --- |
| si*GRP78-1* | GCUCUCUGGUGAUCAAGAUTT | AUCUUGAUCACCAGAGAGCTT |
| si*GRP78-2* | GCAUCAAGCAAGAAUUGAATT | UUCAAUUCUUGCUUGAUGCTT |
| si*GRP78-3* | GGUGGGCAAACAAAGCAUTT | AUGUCUUUGUUUGCCCACCTT |
| si*GRP78-NC* | UUCUCCGAACGUGUCACGUTT | ACGUGACACGUUCGGAGAATT |
