# Supplementary material for: Pyrotinib targeted EGFR/GRP78 mediated cell apoptosis in high EGFR gene copy number gastric cancer
Source: J Exp Clin Cancer Res. 2025 Aug 19;44:245. doi: 10.1186/s13046-025-03485-6 (PMC12363034; doi:10.1186/s13046-025-03485-6)
Supplement: Supplementary file 2 — Supplementary Material 2 [file 13046_2025_3485_MOESM2_ESM.docx]

**Supplementary Figure**


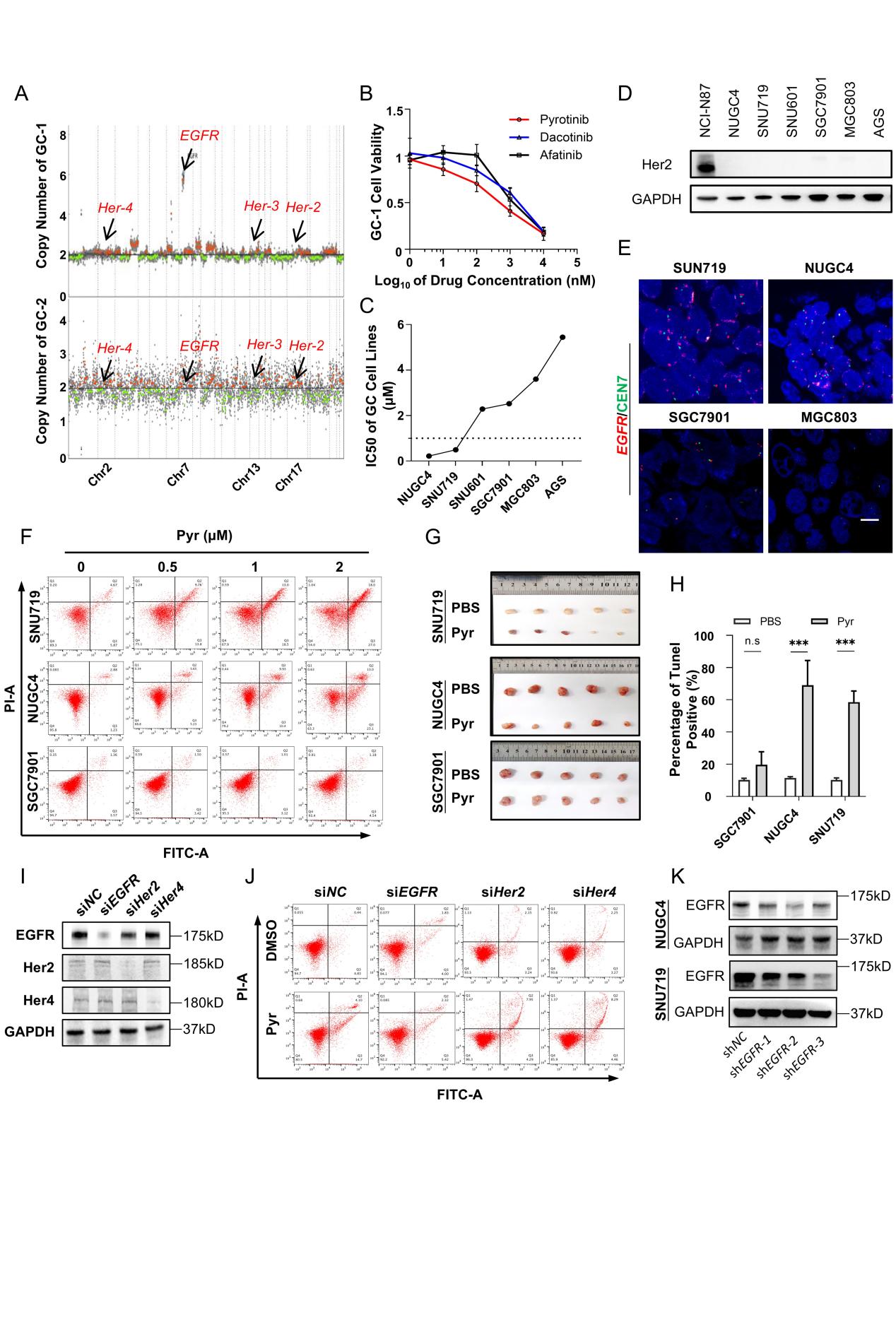


**Supplementary Figure. 1 In EGFR-amplified gastric cancer models, pyrotinib exerts anti-tumor activity by blocking EGFR-mediated signaling cascades.** **A**. Copy number profiles of two primary gastric cancer (GC-1 and GC-2) highlighted gains at HER1/EGFR, HER2, HER3 and HER4. Copy number rations per amplicon were plotted with each individual amplicon represented as a single dot. **B**. GC-1 cells viability was measured by CCK-8 assay followed treatment with indicated concentrations of pyrotinib, dacomitinib, afatinib for 48 h. Data represent three independent experiments. **C**. The IC50 of pyrotinib in 6 GC cells. **D**. Her2 expression of NCI-N87, NUGC4, SNU719, SNU601, SGC7901, MGC803, AGS was testified by western blot. **E**. Fluorescence in situ hybridization (FISH) analysis showed the EGFR status in 4 GC cell lines derived xenograft. Red: EGFR; Green: CEN7. **F**. EGFR-high CN cell (SNU719, NUGC4) and EGFR-low CN cell (SGC7901) were stained with annexin V-FITC/PI and analyzed by flow cytometry followed treatment with increased concentration of pyrotinib for 48 h. **G**. The image of xenograft of SNU719, NUGC4, and SGC7901 treated with PBS or pyrotinib (1 μM) respectively (n = 5). **H**. Histograms showed the TUNEL positive percentage in xenograft tumor tissues from indicated models treated with PBS or pyrotinib (1 μM). **I**. Western blotting verified the expression of EGFR, HER2, HER4 in SNU719 cells transfected with si*N*C, si*EGFR*, si*HER2* and si*HER4* plasmids respectively. **J**. Apoptosis rates of SNU719 cells are inspected by flow cytometry after transfection with respective plasmids and treatment with DMSO or pyrotinib (1 μM). **K**. Knockdown of EGFR expression with sh*RNA* in EGFR-high CN cells (SNU719 and NUGC4) was testified by western blot.


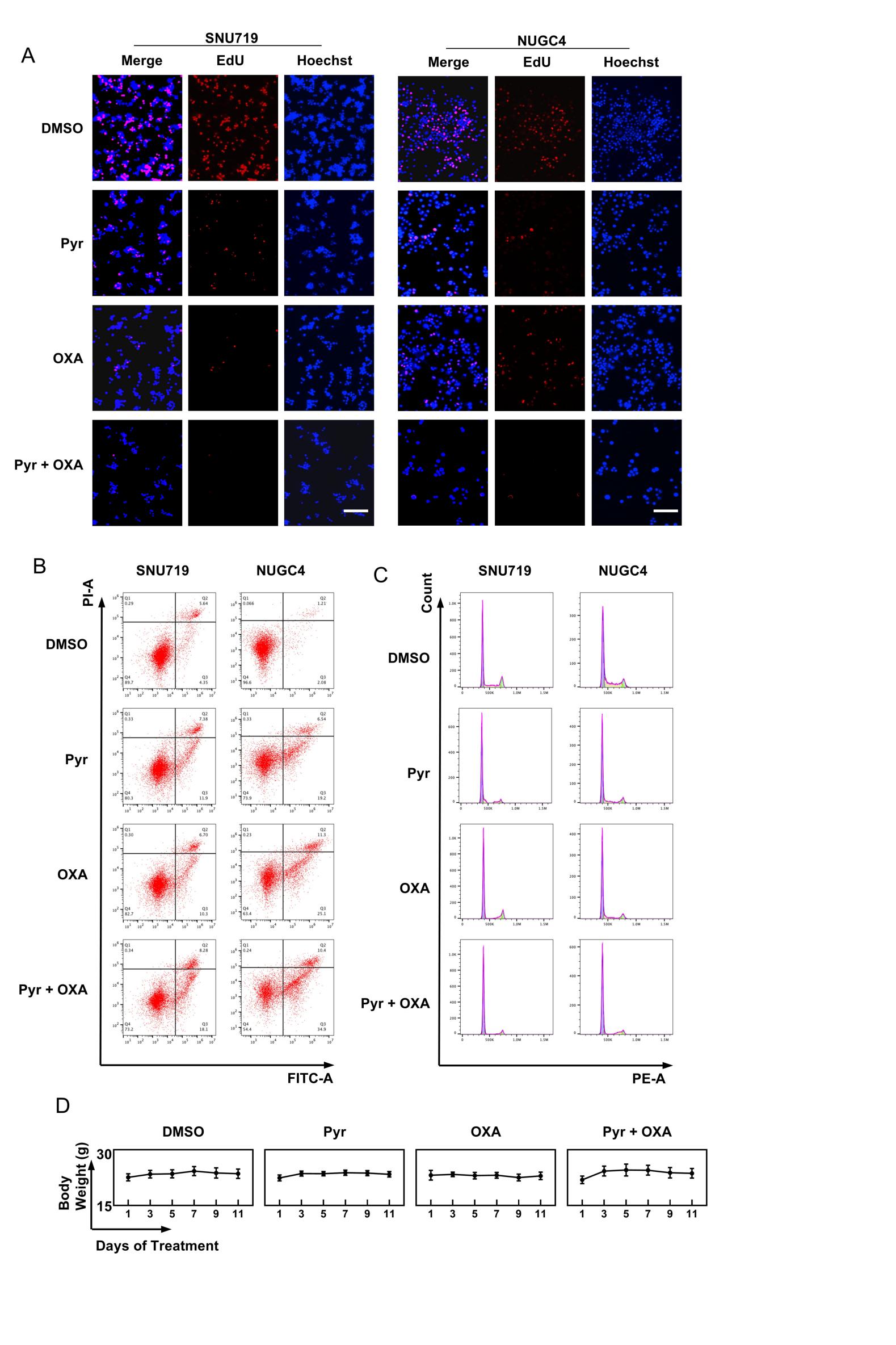


**Supplementary Figure. 2** **The synergistic effect of pyrotinib plus oxaliplatin in EGFR-driven gastric cancer originates from promoting proliferation, inhibiting apoptosis, and arresting cell cycle.** **A**. Representative images of EdU staining in SNU719 and NUGC4 cells treated with DMSO (1 µM), or Pyr (1 µM), or/and OXA (1 μg/ml) for 24 h. Bars, 100 μm. **B**. Annexin V-FITC/PI staining showed the apoptotic cells experienced the indicated treatment. **C**. The cell cycle distributions of SNU719 and NUGC4 cells were determined by flow cytometry after the indicated treatment. **D.** Body weight was measured every 2 days after treatment.


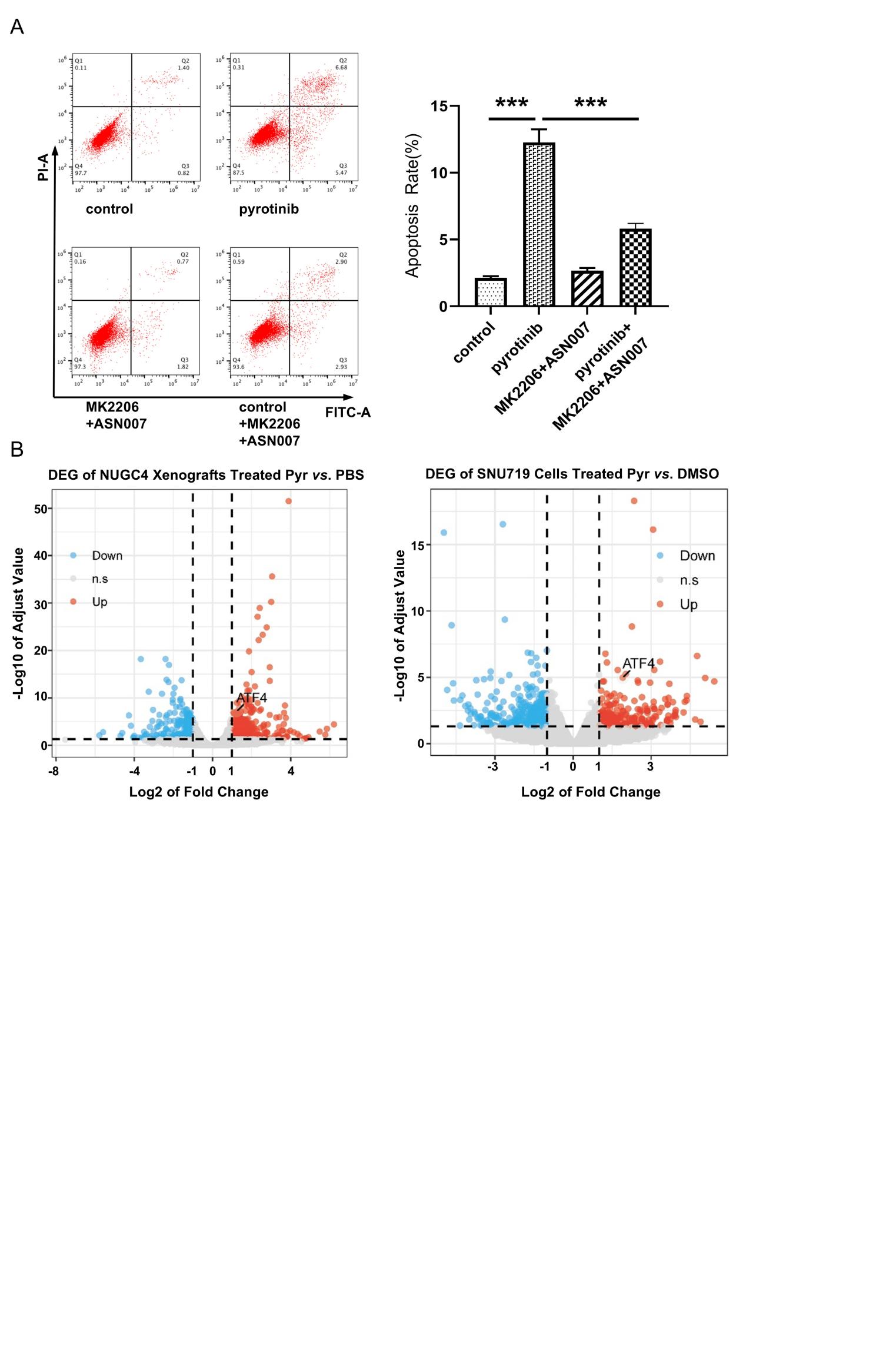


**Supplementary Figure. 3 Differential gene expression analysis in transcriptomics.** **A**. Annexin V-FITC/PI staining showed the apoptotic cells experienced the indicated treatment of pyrotinib, MKN2206 (1 µM)/ASN007 (2 nM) or their combination for 24 h. **B**. Volcano plots displayed RNA sequenced differentially expressed gene (DEG) of NUGC4 xenografts (left pannel) treated with Pyr vs. PBS and SNU719 cells (right pannel) treated with Pyr vs. DMSO.


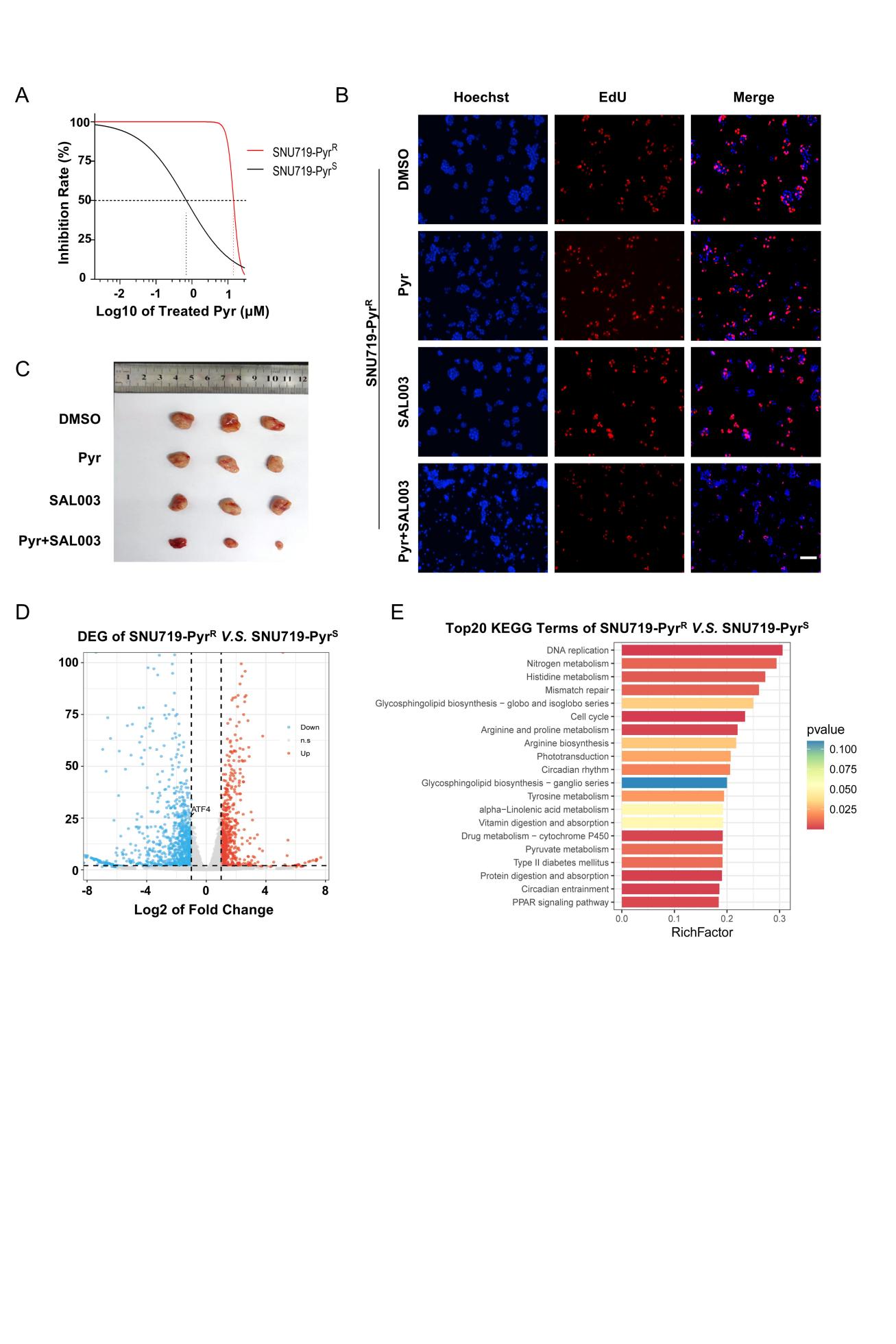


**Supplementary Figure. 4** **Increased p-eIF2α levels reverse pyrotinib acquired resistance.** **A**. Inhibition rates and IC50 values of Pyr in SNU719-Pyr^S^ and SNU719-Pyr^R^ cells. **B**. Representative images of EdU staining in the SNU719-Pyr^R^ cells treated with Pyr (1 µM), or/and SAL003 (10 µM) for 24 h. Bars, 50 μm. **C**. Tumor excised from SNU719-Pyr^R^ cells xenograft tumor models administrated with indicated treatments. **D.** Volcano plots displayed RNA sequenced differentially expressed gene (DEG) of SNU719-Pyr^R^ vs. SNU719-Pyr^S^. **E.** Top 20 enriched KEGG pathways in SNU719-Pyr^R^ vs. SNU719-Pyr^S^.


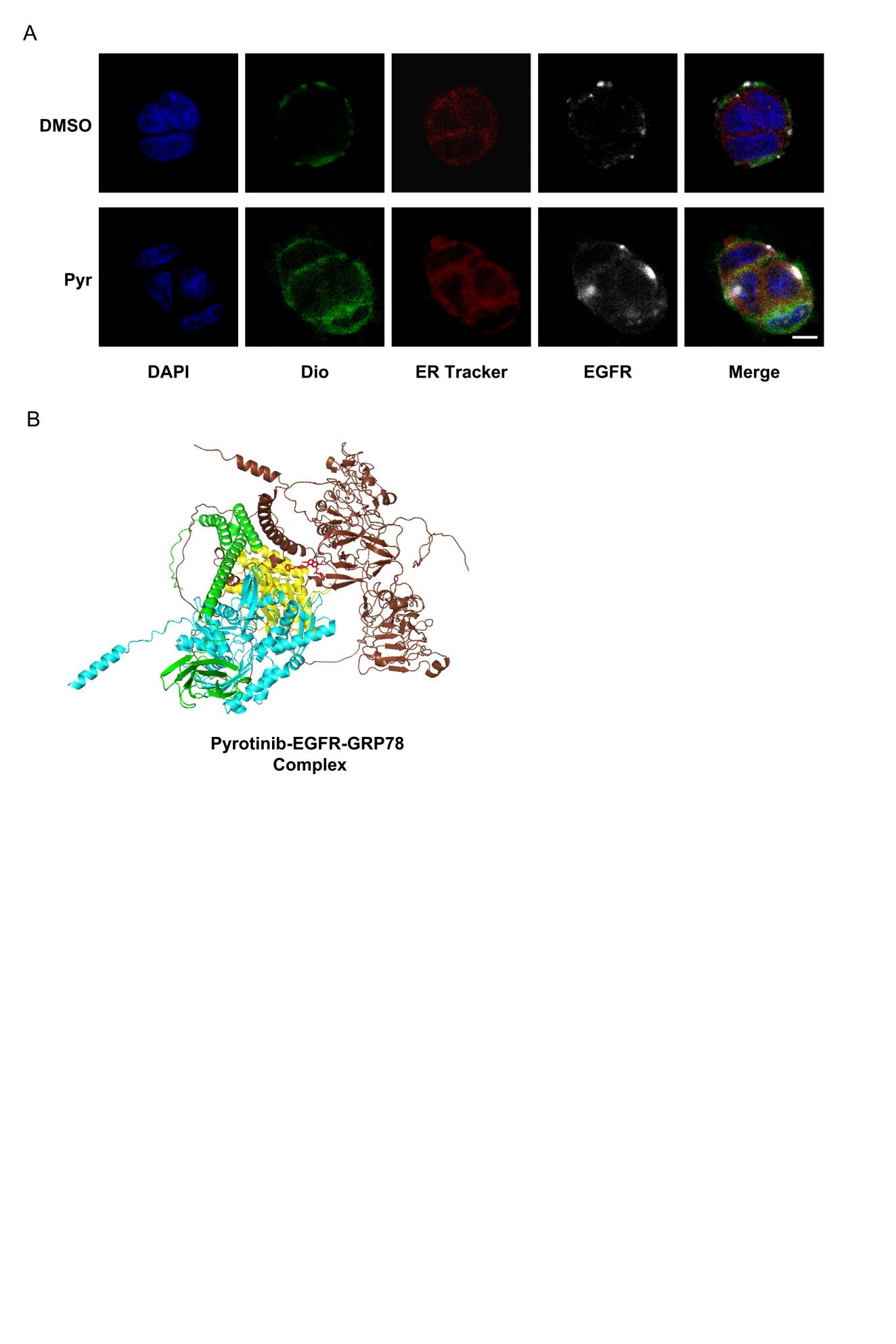


**Supplementary Figure. 5 Pyrotinib induced EGFR translocated from cell membrane to ER. A.** Immunofluorescence analysis of DAPI (nucleus, blue), Dio (cell membrane, green), ER-tracker (red), and EGFR (white) in SNU719 cells treated with Pyr (1 μM) or DMSO for 48 h. Scale bar, 10 µm. **B.** 3D structures for the pyrotinib-induced EGFR-GRP78 complex predicted by GRAMM docking. (Red: pyrotinib; Brown: EGFR; Yellow: TK domain of EGFR; Blue: GRP78; Green: C-terminal of GRP78)

**
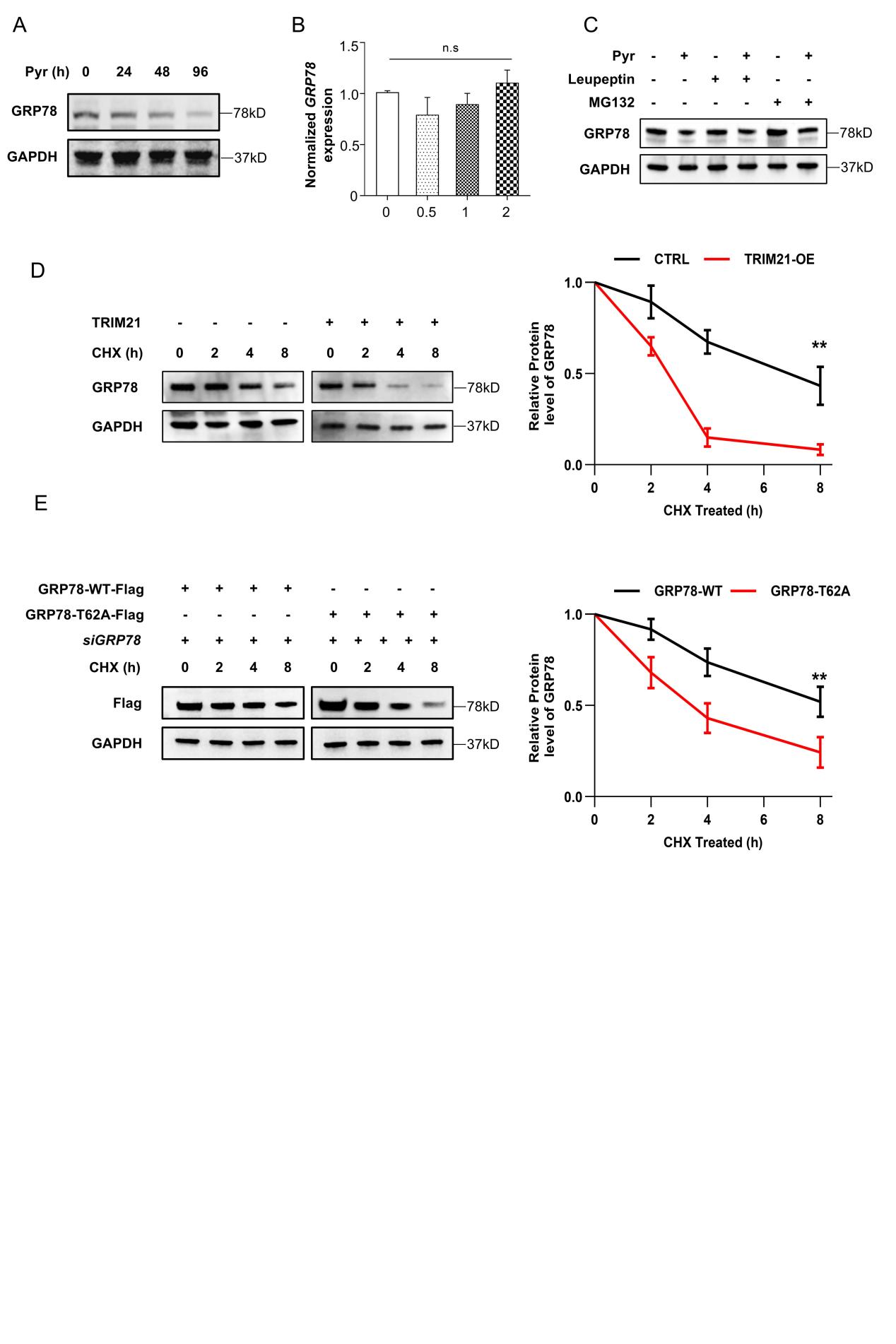
**

**Supplementary Figure. 6 Pyrotinib enhances oxaliplatin chemosensitivity through GRP78 inhibition.** **A**. Western blot analysis of GRP78 protein levels in SNU719 cells treated with Pyr (1 μM) for indicated time. **B**. The GRP78 mRNA in SNU719 cells treated with Pyr (1 μM) at increasing concentrations (0, 0.5, 1, and 2 μM) for 24 hours. **C**. SNU719 cells treated with or without Pyr (1 μM) for 24 h as well as ubiquitin inhibitor MG132 (10 μM) or leupeptin (100 μM) for 6 h. **D.** CHX (100 μg/mL) time-course assays in SNU719 cells revealed accelerated degradation of GRP78 under over-expressing TRIM21 compared to control. **E.** CHX (100 μg/mL) time-course assays in SNU719 cells expressing WT GRP78-Flag or T62A mutant revealed accelerated degradation of the T62A variant.


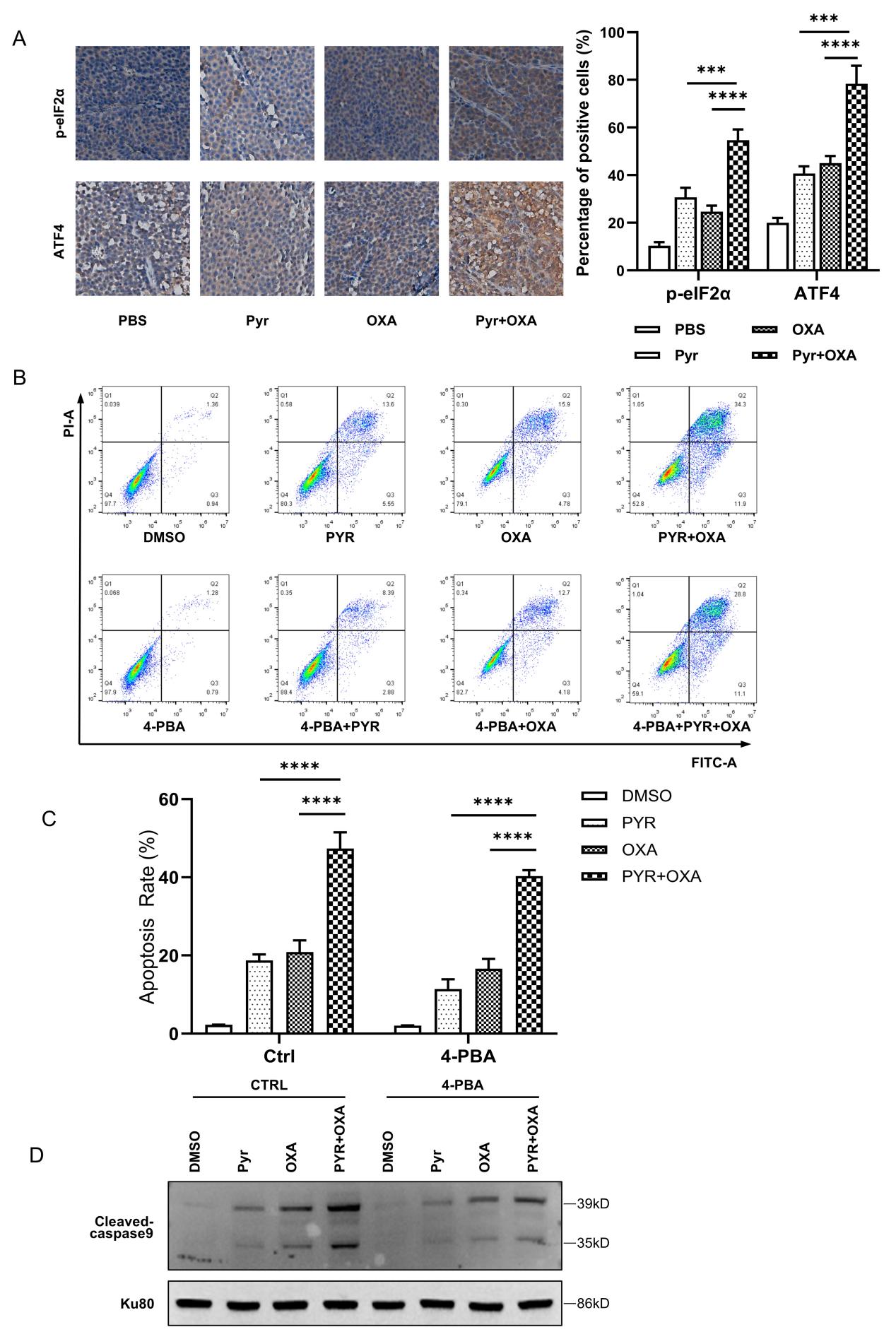


**Supplementary Figure. 7 Pyrotinib-mediated GRP78 suppression enhances oxaliplatin chemosensitivity. A.** Representative immunohistochemical staining of phosphorylated eIF2α (p-eIF2α) and ATF4 in xenograft tumor tissues excised from indicated mice. Bars, 100 μm. **B.** Apoptosis rates of SNU719 cells are inspected by flow cytometry after 48h of indicated treatment with: DMSO, pyrotinib (1 μM), oxaliplatin (2.5 μM), 4-PBA (10 μg/ml) or their respective combinations. **C.** Quantification of apoptosis rates of indicated treatement. **D.** Western blot analysis of cleaved-caspase9 protein levels in SNU719 cells treated with DMSO, pyrotinib (1 μM), oxaliplatin (2.5 μM), 4-PBA (10 ug/ml) or their respective combinations for 48h.
